# Identification of deleterious non-synonymous single nucleotide polymorphisms in the mRNA decay activator ZFP36L2

**DOI:** 10.1080/15476286.2024.2437590

**Published:** 2024-12-13

**Authors:** Betül Akçeşme, Hilal Hekimoğlu, Venkat R. Chirasani, Şeyma İş, Habibe Nur Atmaca, Justin M. Waldern, Silvia B.V. Ramos

**Affiliations:** a Program of Genetics and Bioengineering, Faculty of Engineering and Natural Sciences, International University of Sarajevo, Ilidža/Sarajevo, Bosnia and Herzegovina; b Hamidiye School of Medicine, Department of Basic Medical Sciences, Division of Medical Biology, University of Health Sciences, Üsküdar/İstanbul, Turkey; c Institute of Health Sciences, İstanbul University, Fatih/İstanbul, Turkey; d Biochemistry and Biophysics Department, School of Medicine, University of North Carolina, Chapel Hill, NC, USA; e Biochemistry and Biophysics Department, R. L. Juliano Structural Bioinformatics Core, School of Medicine, University of North Carolina, Chapel Hill, NC, USA; f Department of Molecular Biotechnology, Division of Bioinformatics, Turkish-German University, Beykoz/İstanbul, Turkey; g Department of Medical Biology, Faculty of Medicine, Ondokuz Mayıs University, Atakum/Samsun, Turkey; h Biology Department, University of North Carolina, Chapel Hill, NC, USA

**Keywords:** Zinc finger protein 36 like 2, non-synonymous single nucleotide polymorphisms (nsSNPs), deleterious effect, RNA binding protein, Adenine Uridine Rich Elements (AREs)

## Abstract

More than 4,000 single nucleotide polymorphisms (SNP) variants have been identified in the human *ZFP36L2* gene, however only a few have been studied in the context of protein function. The tandem zinc finger domain of ZFP36L2, an RNA binding protein, is the functional domain that binds to its target mRNAs. This protein/RNA interaction triggers mRNA degradation, controlling gene expression. We identified 32 non-synonymous SNPs (nsSNPs) in the tandem zinc finger domain of ZFP36L2 that could have possible deleterious impacts in humans. Using different bioinformatic strategies, we prioritized five among these 32 nsSNPs, namely rs375096815, rs1183688047, rs1214015428, rs1215671792 and rs920398592 to be validated. When we experimentally tested the functionality of these protein variants using gel shift assays, all five (Y154H, R160W, R184C, G204D, and C206F) resulted in a dramatic reduction in RNA binding compared to the WT protein. To understand the mechanistic effect of these variants on the protein/RNA interaction, we employed DUET, DynaMut and PyMOL to investigate structural changes in the protein. Additionally, we conducted Molecular Docking and Molecular Dynamics Simulations to fine tune the active behaviour of this biomolecular system at an atomic level. Our results propose atomic explanations for the impact of each of these five genetic variants identified.

## Introduction

The post-transcriptional control of mRNA is one of the most important regulators of gene expression. The Zinc Finger Protein 36 family tightly controls post-transcriptional processes by binding to specific target mRNAs that contain adenine-uridine-rich (ARE) elements [1]. The Zinc Finger Protein 36-like 2 (ZFP36L2) is a member of this small family of proteins, which triggers the degradation and/or inhibition of translation of target transcripts after binding to AREs preferentially located in the 3’-untranslated regions (UTRs). ZFP36L2 is involved in the regulation of many different functions, such as mouse female infertility [2–4], erythroid differentiation [5,6], cell proliferation [7], T cell modulation [8,9], thyroid function [10] and muscle development [11]. Additionally, ZFP36L2 has been found to be associated with several cancers, including acute lymphoblastic leukaemia [12], pancreatic cancer [13], and oesophageal squamous cell carcinoma [14] among others.

The three ZFP36 family members (ZFP36, ZFP36L1 and ZFP36L2) were originally described as 12-O-tetradecanoylphorbol-13-acetate (TPA) inducible sequence 11 (TIS11) [15]. TIS11 family was described as composed of three members: TIS11 (ZFP36, tristetraprolin (TTP), Nup475, GOS24), TIS11b (Berg36, ERF-1, ZFP36L1, BRF-1) and TIS11d (ZFP36L2, ERF-2, BRF-2). A fourth family member, ZFP36L3, is restricted to rodents and is expressed in the yolk sac and placenta [16]. ZFP36L3, unlike the other members, is exclusively detected in the cytoplasm and does not shuttle to the nucleus [17]. The ZFP36 proteins have a unique tandem CCCH repeat located in their central part, which is composed of two CCCH zinc fingers spaced 18 amino acids apart. The three cysteines and one histidine coordinate the zinc ion, maintaining the three-dimensional structure of each zinc finger domain. The integrity of both zinc fingers is required for binding to their target transcripts; disruption of either one of the zinc fingers is sufficient to abolish the RNA binding capability [18–20]. Upon binding, the bound transcript is deadenylated and destabilized [21], ultimately resulting in reduced protein production from these targeted mRNAs. The tandem zinc finger domain is the functional RNA-binding domain (represented in blue in supplementary Figure S1) that characterizes this family of proteins. As shown in supplementary Figure S2, 60% of the amino acids (46/79) that compose the tandem zinc finger domain are identical between the three human orthologs, however there is minimal amino acid sequence homology outside this domain.

Interestingly, in biochemical assays, all three family members can bind to the same iconic targets, such as the multiple AREs of tumour necrosis factor-alpha (TNF-α) and granulocyte macrophage colony-stimulating factor (GM-CSF) mRNA [20,22]. However, knockout mice of each of these proteins lead to dramatically different phenotypes. ZFP36-KO leads to an inflammatory syndrome resembling lupus and rheumatoid arthritis [23,24]. ZFP36L1-KO is lethal due to chorioallantoide fusion defects [25]. ZFP36L2-KO leads to severe pancytopenia of all haematopoietic cell lines and in death at the second week of life [5,26]. These distinct phenotypes suggest that despite potential-binding redundancy, they clearly have different physiological roles *in vivo*.

To further understand how structural disturbances in the tandem zinc finger domain can affect the RNA-binding activity, we investigated the single nucleotide polymorphisms found in the tandem zinc finger domain of ZFP36L2. Single nucleotide polymorphisms (SNPs) are the most common alterations in the human genome [27]. Although many of SNPs are neutral, some variants do affect gene expression or the function of the translated proteins. Approximately 4,000 known variants, including insertions, deletions, and SNPs, have been found in the *ZFP36L2* gene, however only a few have been tested for functional consequences. Here, we investigate the possible deleterious effects of non-synonymous coding SNPs (nsSNPs) located in the tandem zinc finger domain of ZFP36L2 using *in-silico* predictions followed by experimental validation. We identified 32 nsSNPs in the tandem zinc finger domain of ZFP36L2. Among them, 5 of 32, were bioinformatically predicted to be highly deleterious nsSNPs. Upon experimental evaluation, all five nsSNPs predicted to affect ZFP36L2 function resulted in a dramatic reduction in the RNA binding ability compared to the WT protein. In contrast, 3 of 32 nsSNPs predicted to be non-deleterious did not affect RNA binding.

## Methods

### Retrieval of ZFP36L2 gene mutations

Human *ZFP36L2* gene information was retrieved from the National Center for Biotechnology Information (NCBI) dbSNP database. Missense mutations were filtered, and 514 non-synonymous coding single nucleotide polymorphisms (nsSNPs) were used for further screening. The dbSNP information includes SNP ID, protein accession number (NP), mRNA accession number (NM), location, changes in the residue, and worldwide minor allele frequency (MAF). The FASTA sequence of the protein was retrieved from the UniProt database (UniProt ID: P47974). The structural domain of ZFP36L2 protein was obtained from the Protein Data Bank (PDB) (PDB ID: 1RGO).

### Prediction of nsSNPs pathogenicity

PredictSNP is a consensus classifier (https://loschmidt.chemi.muni.cz/predictsnp) that predicts functional effect of the nsSNPs collected from the dbSNP database. PredictSNP combines six of the best prediction tools (MAPP, PhD-SNP, PolyPhen-1, PolyPhen-2, SIFT, and SNAP) to improve prediction performance [28]. Thus, PredictSNP integrates these six tools to generate its own prediction, in addition to the individual results derived from each of these six tools. MAPP is a web server that predicts the impact of mutations on protein function based on physicochemical properties and alignment scores [29]. PhD-SNP is another web server used to predict deleterious human SNPs based on a binary classifier field [30]. PolyPhen-1 is a tool to predict the impact of missense mutations on the structure and function of a human protein using straightforward physical and comparative considerations, whereas PolyPhen-2 differs from the previous version in terms of the set of predictive features, alignment pipeline, and the method of classification (Naive Bayes classifier) [31,32]. SIFT is an online server that predicts whether an amino acid substitution affects protein function based on alignment score and the physical properties of amino acids [33]. SNAP applies a neural network approach to identify if a mutation is non-neutral (impact on function) or neutral (no impact) by incorporating evolutionary information, predicted aspects of protein structure, and other relevant information [34]. Each tool uses different parameters and pipelines to identify deleterious mutations. We used these programs in a combined fashion to enhance the reliability of our predictions.

SNPs&GO is a Support Vector Machine (SVM) based predictor that integrates Gene Ontology (GO) into its methodology. It uses the protein sequence profile and functional information as input features for the SVM. If the probability score is > 0.5, the mutation is considered disease-related, if not, it is categorized as neutral [35].

MutationAssessor predicts the functional impact of missense mutations in cancer or polymorphisms based on the evolutionary conservation of the affected amino acid using multiple sequence alignment of proteins from the same family. It defines the functional impact of a mutation as high, medium, low, or neutral. If the impact of a variant is predicted as functional, it is then classified as high or medium. If a variant is defined as neutral or low, then it is predicted as non-functional repercussion [36].

### Evolutionary conservation analysis

ConSurf (http://consurfdb.tau.ac.il/) is a server that uses the estimated evolutionary conservation of amino acid positions in a protein based on the phylogenetic relations between homologous sequences by using either an empirical Bayesian method or a maximum likelihood (ML) method [37–42]. The ConSurf server calculates the evolutionary rates of a particular position of DNA/RNA/protein by performing evolutionary analyses based on phylogenetic ranking of homologous sequences. It retrieves non-redundant PDB entries for analyses and utilizes the Uniprot reference clusters (UniRef) database to find homologous sequences in different organisms. Multiple sequence alignment (MSA) and phylogenetic tree construction steps are included to find the homology levels of sequences. Assessment of the evolutionary conservation of an amino acid can provide information about its structure and function; the more conserved a given amino acid is, the more likely it plays a relevant role in the structure and function of a given protein domain. ConSurf scores amino acids at each position ranging from 1 to 9, based on their evolutionary conservation, where 1 is the most highly variable, 5 is of intermediate conservation, and 9 is the most highly conserved position.

### Experimental procedures

Plasmid Constructs – human ZFP35L2-WT (NM_006887) was inserted into the pCMV6 vector in-frame with a Flag sequence at the carboxy terminal end of the protein, as previously described [43]. Each nsSNP, namely: Y154H (rs375096815), R160W (rs1183688047), R184C (rs1214015428), G204D (rs1215671792), C206F (rs920398592), S185N (rs759784530), T187S (rs1274850041) and D210E (rs201694029) were created by PCR mutagenesis and inserted in the pCMV6 vector using the same restriction enzyme sites as the WT (GenScript). Thus, all constructs contain the same CMV promoter and *Bovis* growth hormone 3’UTR. For the fluorescence analysis, the eGFP sequence (KX510271.1) preceded by a favourable Kozak sequence was inserted at the amino terminus of ZFP36L2 proteins in-frame (GenScript). All constructs were verified and re-sequenced in the lab.

Cell Culture, Transfections, and Protein Extracts – HEK 293 cells (American Type Culture Collection CRL-3216) were maintained in DMEM medium supplemented with 10% foetal bovine serum, penicillin (100 units/mL), and streptomycin (100 μg/mL). Transient transfection of 2 × 10^6^ cells seeded in a 100 mm plate with different human *ZFP36L2* plasmids were performed using Lipofectamine 2000 (Life Technologies) according to the manufacturer’s protocol [43]. The transfection mixture containing 1 µg of DNA for each construct was incubated with the cells for 12–18 hours and then replaced with fresh medium for a further 24-hour incubation, after which the cells were lysed for protein extraction or analysed on a fluorescent microscope for subcellular localization.

Immunoblot Analysis – Protein samples were quantitated by Bradford assay (Bio-Rad). Five μg of protein per lane was loaded on a 10% Tris-Glycine polyacrylamide gel and electrophoresed under denaturing conditions. The proteins were transferred from the gel to a nitrocellulose membrane using the Trans-Blot® TurboTM System (Bio-Rad). The membrane was blocked for 1 h with 5% non-fat milk (w/v) in a Tris-buffered saline solution containing 0.1% TWEEN (TBS-T). The following primary antibodies were used for protein detection: mouse monoclonal anti-β-actin (GeneTex GT5512), monoclonal anti-DDK (Origen, TA5001, clone OT14C5). The HRP-conjugated secondary antibody was a sheep anti-mouse (Gena-N931V). The HRP signal was developed using the Bio-Rad Western Clarity TM chemiluminescent substrate.

RNA Electrophoretic Mobility Assay – Wild type ZFP36L2 (WT-L2) or ZFP36L2 different SNPs Mutants and mouse Zfp36l2 (C175S, disruption of the first zinc finger domain), or empty vector plasmids were transiently transfected into HEK 293 cells. Then, protein extracts were prepared as described [18]. The protein extracts were incubated for 15 min at room temperature with 0.2 × 10^5^ cpm of ^32^*p*-labeled RNA probe in a final volume of 20 μL containing 10 mm HEPES (pH 7.6), 40 mm KCl, 3 mm MgCl_2_, 0.5 μg/μL heparin, and 60 ng/µL yeast tRNA, as described [18]. The resultant reaction mixtures were applied to 6% nondenaturing acrylamide (37.5:1) gels and subjected to electrophoresis at 150 V for 15 min followed by electrophoresis at 200 V for 90 min in 0.4× Tris-borate/EDTA running buffer. The gels were dried, exposed to film (Carestream BIOMAX MR Film), and developed after 12–20 h of exposure. Gels were also exposed to a PhosporImager screen, scanned using an Amersham Typhon 6 imager and quantified using ImageQuant software (GE Healthcare). The amount of each bound and unbound probe was quantified, and values were normalized to the background of each experiment. Each gel shift assay was repeated four times, using different protein extracts to ensure rigour and reproducibility.

Preparation of RNA Probe for RNA Electrophoretic Mobility Assay – The RNA probe was synthesized with the Riboprobe System-T7 (Promega) using DNA primer sequences immediately downstream from a T7 promoter, as previously described [44]. The RNA probe was body-labeled during the transcription process, which was performed in the presence of [α-^32^P] UTP (3000 Ci/mmol; PerkinElmer). The probe was designed to be around 30 nucleotides including the ARE motif. The synthesized RNA probe was separated from the free nucleotides using Sephadex G50 columns (GE Healthcare Life Sciences) and subsequently electrophoresed on a 16% polyacrylamide urea gel. The probe was purified from excised gel fragments after detection by autoradiography, as previously described [18]. The amount of RNA probe used in each lane of the EMSA was calculated to be ~10 femtomoles. The sequence of the RNA probe contained ARE sequences from the 3’UTR of *Gm-csf*. The corresponding sequence is listed below.

*Gm-csf*: 5’−5’UACCUUAUACUUGAAUUUAUUUAUUUAUUU-3’

Subcellular Localization Analysis by Epifluorescence – For epifluorescence, 1 × 10^6^ HEK 293 cells were transiently transfected in a 35 mm petri dish containing a 10 mm microwell (MatTek corporation, MA) with 500 ng of each eGFP-ZFP36L2 constructs and visualized with a Nikon ECLIPSE Ti2 inverted microscope with a spinning disk for live cell imaging.

### Effects of the amino acid substitution on protein structure

#### Using bioinformatic tools

Given that protein conformation is an important factor that affects the function and biological activity of molecules, the effects of a single amino acid change on protein structure were evaluated using three tools: DUET, DynaMut and PyMOL with the goal of gaining insight into the structural consequences of the five predicted deleterious mutations (Y154H, R160W, R180C, G204D, and C206F). The FASTA sequence of the protein was retrieved from the UniProt database (UniProt ID: P47974) and the structural domain of the ZFP36L2 protein was obtained from the Protein Data Bank (PDB) (PDB ID: 1RGO). We systematically introduced each mutation into the wild-type (WT) ZFP36L2 and generated the corresponding mutant structures. Each novel protein structure resulted in a different folding energy which was compared to that of the WT. The main difference is that each of these tools generate final predictions based on different sets of parameters, and thus may lead to distinct final outputs. DUET and DynaMut are tools which predict the effect of mutations on protein function through the determination of the stability of interactions with other molecules including proteins, nucleic acids, and small molecules. DUET predicts the effect of a single point mutation using an integrated computational approach that pools the results of mCSM and Site-Directed Mutator (SDM) in an optimized combined prediction by consolidating the results of these two methods using Support Vector Machines (SVMs) trained with Sequential Minimal Optimization [45]. SDM is an optimized knowledge-based approach that relies on amino acid propensities derived from environment-specific substitution tables for homologous protein families feeding a statistical potential energy function [45–47]. DynaMut combines Normal Mode Analysis (NMA) methods and graph‐based signatures by sampling protein conformations and assesses the impact of mutations on protein dynamics and stability resulting from vibrational entropy changes [48,49]. PyMOL (PyMOL Molecular Graphics System, Version 2.0 Schrödinger, LLC.) is a versatile molecular visualization and analysis tool. Utilizing PyMOL’s *mutagenesis* tool, we introduced each mutation into the wild-type (WT) ZFP36L2 structure and generated corresponding mutant structures to subsequently analyse the structural impact of the mutations, such as alterations in hydrogen bonding patterns. Specifically, we assessed hydrogen bonds induced by WT and mutant structures to elucidate potential disruptions in protein stability and function.

Additionally, we have analysed the WT protein, the five protein variants predicted to be deleterious (Y154H, R160W, R184C, G204D and C206F) and three predicted to be non-deleterious (S185N, T187S and D210E using AlphaFold 3.0. The confidence scores for these predictions were in the highest interval (plDDT > 90), especially when comparing critical regions.

#### Performing experiments using cellular temperature shift assay (CETSA)

Twenty-four hours after the transfection mixture was removed, HEK 293 cells overexpressing the WT or the protein variants Y154H, R184C, and C206F were harvested by trypsinization and washed three times with 10 mL of cold PBS. Each cell sample was counted and adjusted to 1 × 10^7^ cells per mL. The same number of cells (around 20 × 10^6^ cells) was dispensed in 50 μL in each PCR tube. Cells overexpressing either WT or each of the protein variants Y154H, R184C and C206F were subject to six different temperatures: RT, 40°C, 43°C, 48°C, 50°C, and 55°C for 6 min using a Thermal cycler and then cooled down on ice for 6 more minutes. Lysis buffer (50 μL) was added to each sample and the mixture was incubated for 10 min with continuous rotation at 4°C. After lysis, the samples were subjected to centrifugation at 10,000 × g at 4°C for 30 min. The supernatant containing the soluble protein fraction was carefully collected and transferred to a new tube. These samples were then subsequently analyzed by sodium dodecyl sulphate polyacrylamide gel electrophoresis (SDS-PAGE), followed by Western blotting with a Flag antibody.

### Molecular docking analysis

#### Preparation of molecules for docking

The tandem zinc finger domain of ZFP36L2 bound to UUAUUUAUU, the 9-mer, experimentally resolved PDB ID: 1RGO [50] obtained from PDB was used in docking and other structure-based analysis. It was also used as positive control for the docking analysis. Another PDB file was generated using a mutant version of the protein (C169F) known to destroy the normal conformation of the first zinc finger domain;, and because this mutation is also known to abolish RNA binding, it was used as a negative control. We used the UCSF Chimera tool-rotamer function to obtain each corresponding structural induced model for each identified mutation (Y154H, R160W, R184C, G204D, and C206F) before testing the docking. UCSF Chimera is a software that uses interactive visualization for the analysis of molecular structures and related data, such as density maps, trajectories, and sequence alignments [51].

#### Docking analysis of wild-type and ZFP36L2 protein variants

Molecular docking analysis of wild-type and mutant versions of the tandem zinc finger domain from ZFP36L2 with 9-mer oligomers was performed using docking tools [52,53]. HADDOCK2.4 is an integrative platform for the modelling of biomolecular complexes that performs docking using a data-driven approach. The docking encompasses three stages: rigid body docking, semi-flexible refinement, and water refinement. It supports a large variety of input data such as multi-component assembles of proteins, peptides, small molecules, and nucleic acids. HDOCK is a docking tool based on a hybrid algorithm of template-based modelling and *ab initio* free docking for protein–protein and protein-DNA/RNA docking [54]. For HADDOCK2.4 the parameters applied were as follows: binding amino acid residues of WT–tandem zinc finger domain of ZFP36L2, corresponding to the positions 153, 154, 156, 157, 158, 160, 161, 168, 169, 170, 171, 173, 175, 176, 189, 191, 192, 193, 194, 195, 196, 198, 199, 207, 208, 211, and 213 were docked with its respective binding residues of the 9-mer. For the proteins, we first generate PDBs for each identified nsSNP. We performed the molecular docking first by introducing the substitute amino acid (Y154H, R160W, R184C, G204D, and C206F) into the tandem zinc finger domain structure using UCSF Chimera. Thus, each PDB corresponded to a novel structured mutant protein. One extra PDB was generated, by introducing a mutation known to disrupt protein/RNA interaction [22]. The goal of this conformational mutant was to create a protein known to abolish the RNA interaction and expected to drastically affect the molecular docking. We performed the molecular docking of the WT and mutants to the 9-mer ARE (UUAUUUAUU) using HADDOCK2.4 and HDOCK tools. HADDOCK scoring function consists of a linear combination of various energies and buried surface areas resulting in a final score. The docking poses of the tandem zinc finger domain of ZFP36L2 mutants and RNA oligonucleotide were visualized using BIOVIA Discovery Studio 2021. Each structural mutant protein bound to the RNA oligonucleotide, 9-mer (UUAUUUAUU), was obtained and subsequently compared to wild-type tandem zinc finger domain of ZFP36L2 bound to the 9-mer and whose structure was obtained experimentally [50].

#### Molecular dynamics simulations

Molecular dynamic simulations of both wild-type (WT) and variant ZFP36L2 proteins (Y154H, R160W, R180C, C206F, and G204D) were conducted using the GROMACS 2020.3 software package [55] employing the AMBER99SB force field [56]. Each system was solvated in a cubic box of TIP3P water molecules, ensuring a minimum distance of 10 Å between the protein and the box edges. The system was neutralized by adding 0.15 M NaCl ions and maintained under physiological conditions (310 K temperature, 1 bar pressure) using NVT (constant number of particles, volume, and temperature) and NPT (constant number of particles, pressure, and temperature) ensembles. Prior to production molecular dynamics simulation runs, the systems underwent equilibration simulations for 10 ns with a time step of 2 fs. During equilibration, positional restraints were applied to the protein backbone atoms to relax the solvent molecules around the protein. The LINCS algorithm [57] was employed to constrain hydrogen bonds, allowing for a stable integration of equations of motion. Long-range electrostatic interactions were treated using the particle mesh Ewald (PME) method [58] with a cut-off of 12 Å. Following equilibration, the systems were subjected to production of molecular dynamics for 1000 ns for each system to explore their dynamical behaviour and obtain statistically significant data. Trajectories were saved at regular intervals for subsequent analysis. We conducted three replicates of molecular dynamics simulations for each of the ZFP36L2 variants to ensure the reliability and reproducibility of our findings.

#### Hydrogen Bond Analysis

The hydrogen bonding patterns in both WT and mutant ZFP36L2 proteins were analysed using the *gmx hbond* tool. Hydrogen bonds were defined based on distance and angle criteria between donor and acceptor atoms. A hydrogen bond was considered formed if the distance between the hydrogen and the acceptor atom was less than 3.5 Å and the angle between the donor-hydrogen-acceptor was greater than 120º. The occurrence and stability of hydrogen bonds throughout the simulations were monitored and compared between WT and mutant systems. The 3D structural visualization of average conformations extracted from the molecular dynamics trajectories was performed using the PyMOL Molecular Graphics System (The PyMOL Molecular Graphics System, Version 2.0 Schrödinger, LLC).

## Results

The human *ZFP36L2* transcript is 4,372 nucleotides long and comprises two exons. Its coding gene is in the long arm of chromosome 2. It has only one isoform/transcript that produces a 494 amino acid-long protein [59]. The protein is comprised of a tandem zinc finger domain in which the zinc ions are coordinated by three cysteines and one histidine (supplementary Figure S1). As the initial step of mRNA decay, the tandem zinc finger domain recognizes and binds to the UAUUUAU 7-mer, which is considered the minimal core motif [26]. However, ZFP36 (TTP) seems to preferentially bind to multiple adjacent AREs, classified as ARE class II [19,60].

### Prediction of deleterious nsSNPs using Sequence-based Analysis Tools

The workflow used for our study is schematized in supplementary Figure S3. Five hundred and fourteen SNPs for the *ZFP36L2* gene were retrieved from NCBI dbSNP. Out of 514 nsSNPs, only 32 nsSNPs are in the functional tandem zinc finger domain of ZFP36L2 which is also the only domain with a known three-dimensional structure in PDB. To predict disease-related mutations, these 32 nsSNPs were classified using PredictSNP (supplementary Table 1). PredictSNP is a consensus classifier that combines the tools MAPP, PhD-SNP, PolyPhen-1, PolyPhen-2, SIFT and SNAP to predict the effects of nsSNPs on the protein function.

**Figure 1.**
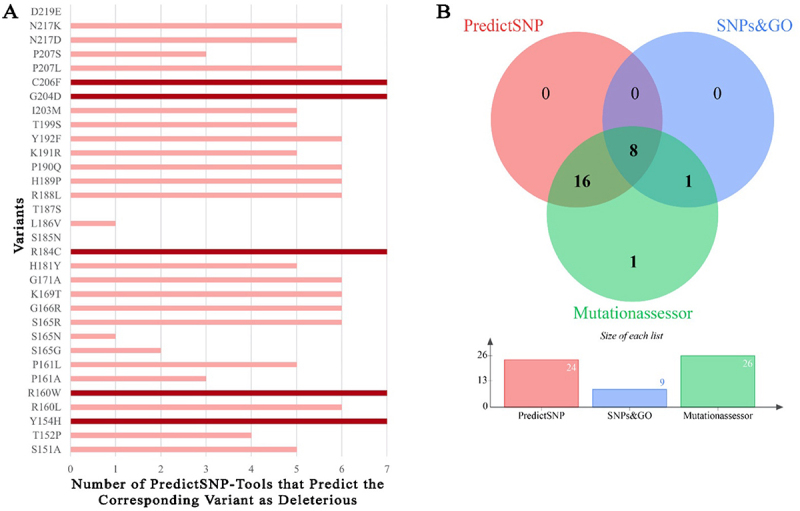
Function predictions of SNP variants in the tandem zinc finger domain of ZFP36L2. (A)List of 32 SNP variants identified by predictSNP-tools (predictSNP, MAPP, phd-snp, PolyPhen-1, PolyPhen-2, SIFT, and SNAP). Five variants (dark red bars) were simultaneously classified as functionally deleterious by all seven classifiers, while the remaining 24 SNOs (light red bars) were predicated to have functional consequence only by six or less tools. The three variants, (no bars) were predicted to be neutral by all seven tools. (B) these 32 SNPs located in the tandem zinc finger domain were further analysed in terms of potential disease association using SNPs%GO and MutationAssessor predictors, venn diagram shows number of common mutations by PredictSNP (single tool), SNPs&GO and MutationAssessor.

Accordingly, five nsSNPs were identified as potential deleterious variants simultaneously by the PredictSNP classification and by six other tools (Figure 1A and supplementary Table 1). The incidence of these five variants in the population is relatively low, with an allele frequency of 0.007% for rs375096815 and 0.003% for rs1183688047, whereas the allele frequencies for rs1214015428, rs1215671792 and rs920398592 were <0.001%. These SNPs would result in the following amino acid substitutions: Y154H, R160W, R184C, G204D, and C206F, respectively.

Additionally, these 32 nsSNPs located in the tandem zinc finger domain were also analysed with SNPs&GO and MutationAssessor to predict mutation pathogenicity (supplementary Table 2 and 3). The common nsSNPs predicted by PredictSNP, SNPs&GO and MutationAssessor resulted in eight nsSNPs classified as disease-related (Figure 1B, Venn diagram intersection). All five nsSNPs, except one (rs1183688047, R160W), shown in Figure 1A are included in the eight nsSNP intersection in Figure 1B. However, this nsSNP, the variant R160W, was assessed as neutral by SNPs&GO with a probability score of 0.438 (supplementary Table 4), whereas the other four nsSNPs (Y154H, R184C, G204D, and C206F) reached probability scores >0.5. Because the other remaining four nsSNPs present in the intersection of Figure 1B were not predicted to be deleterious using the seven distinct bioinformatic classifiers shown in Figure 1A, we decided to focus our subsequent investigation on the five nsSNPs results obtained from all seven PredictSNP-Tools as summarized in Figure 1A and detailed supplementary Tables 1, 2, 3 and 4. Note that, in supplementary Table 4, PredictSNP and PhD-SNP had a better overall accuracy (around 87%) than the other five tools used in Figure 1A.

### Evolutionary conservation analysis of the WT zinc finger domain

It is important to note that the frequency of SNPs tend to be smaller in the *genomic* sequence of conservation. However, SNPs occur randomly and only in one allele, whereas the other allele encodes the WT protein. Using on our ZFP36L2 knock-out mouse model, we noticed that the presence of only one allele is sufficient for normal protein function *in vivo* and no phenotype was observable [26]. Thus, a potential deleterious SNP when simultaneously expressed with the WT *ZFP36L2* allele, in heterozygosity, may not lead to any observable phenotype; thus, it is not subjected to evolutionary conservation pressure.

**Figure 2.**
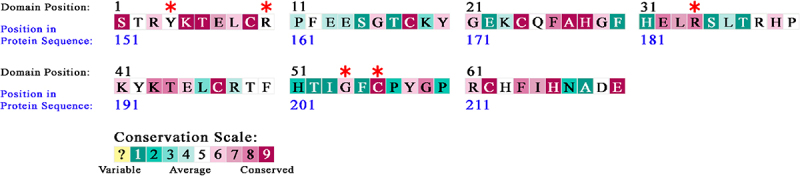
Evolutionary conservation analysis. All amino acids in the tandem zinc finger domain were subjected to evolutionary conservation analysis using ConSurf. The location of the five nsSNPs predicted to be deleterious-namely Y154H, R160W, R184C, G204D, and C206F, and the colour conservation results for the amino acid they substitute are indicated by red asterisks.

However, if a SNP occurs in a location of an amino acid that is highly conserved, the likelihood to have deleterious effect in terms of RNA binding function would be higher. Since the evolutionary conservation of the tandem zinc finger domain most likely corresponds to its critical function, if a SNP occurs at a location of conservation, it would be highly likely to have deleterious effect in terms of RNA binding. Thus, we investigated the evolutionary conservation of the amino acids composing the functional domain of ZFP36L2. In our study, 5,817 homologues were collected from the UNIPROT database using HMMER from the ConSurf server. Of these, 3,690 homologues passed the thresholds (min/max similarity, coverage, etc.), whereas 2,201 of them were CD-HIT unique. The calculations were conducted on 300 hits (query included), sampled from the unique hits. The conservation level of a given position of interest was visualized using a colour scale (Figure 2), ranging from 1 to 9, where 1 is the most highly variable and 9 is the most highly conserved position. Based on this scale, Y154H and R160W variants were found as moderately conserved, whereas variant R184C, G204D and C206F were found to be highly conserved. In summary, we obtained results supporting that three of our five deleterious nsSNP candidates were in an evolutionarily conserved position of the zinc finger domain, with a high conservation score, whereas Y154H and R160W had an average conservation score. However, it is interesting to note that residues 154 and 160 are amino acids that directly interact with the RNA [50].

### Experimental validation of the five Predicted Deleterious nsSNPs Located in the tandem zinc finger domain

**Figure 3.**
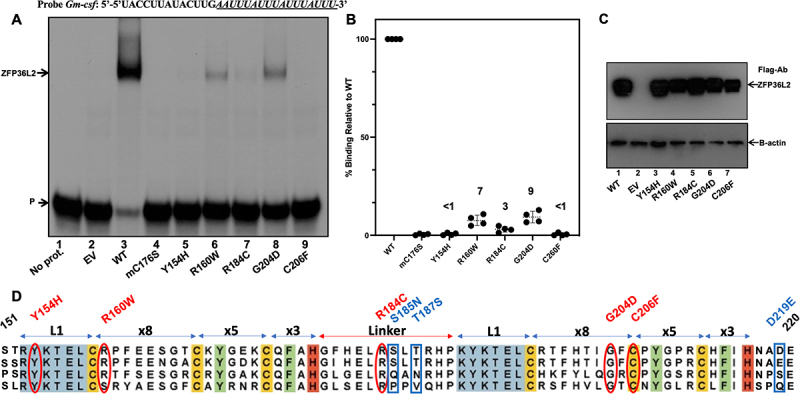
ZFP36L2 protein variants expressing the 5 deleterious nsSNPs were compared to the wt protein ability to bind to RNA. (A) RNA electrophoretic mobility shift assays were performed by incubating 20 µg of protein extracts from HEK 293 cells transfected with empty vector (EV, lane 2) or with a vector expressing the WT or different ZFP36L2 variants (lanes 5 to 9) with 0.2 × 10^5^ cpm of *Gm*-csf probe. Lane 4 corresponds to a mutation in the first zinc finger domain of mouse previously shown to abrogate binding (mC176S). Lanes 5 to 9 correspond to Y154H, R160W, R184C, G204D and C260F, respectively. (B) EMSA were performed with protein extracts from four different transfections and the bound and unbound probe (P) were quantified using a PhosphorImager, hatched lines are means and bars are standard deviation, (C) immunoblotting of protein extracts used in one EMSA were probed with flag antibody. Five µg of protein extracts were loaded per lane and probed with a beta-action (lower panel) or anti-flag antibody (upper panel), (D) alignment of tandem zinc finger of hZFP36L2 (TIS11D), hZFP36L1 (TIS11B), hZFP36 (TTP), and xenopus. The location of each deleterious nsSNPs is illustrated in red and the predicted non-deleterious in blue.

Given that three of our five nsSNP candidates were highly evolutionarily conserved, these three nsSNPs would be more likely to change the protein interaction with its RNA partner. In two nsSNPs, Y154H and R160W, the evolutionary conservation was found to be only ‘moderate’. Additionally, R160W was assessed as neutral in terms of pathogenicity/disease-relatedness using the SNPs&GO with a probability score of 0.438, slightly below 0.5. Thus, we questioned if the comparatively less conserved amino acid substitutions, Y154H and R160W, would effectively be deleterious. To investigate the effects of these protein variants on RNA binding, we used RNA gel shift assays (EMSA). We previously showed that ZFP36L2 binds to *Gm-csf* in a dose-dependent manner with nanomolar affinity [26]. Consequently, we chose the *Gm-csf* ARE containing probe to test the binding. Each lane contains 20 μg of total protein extract of HEK 293 cells overexpressing only one protein variant interacting with the probe. When we tested our protein variants derived from the five nsSNPs predicted to be deleterious and compared with WT-ZFP36L2 binding, it was clear and consistent that Y154H and C206F were essentially unable to bind to RNA and resulted in less that 1% binding (Figure 3A, lanes 5 and 9; Figure 3B). Interestingly, the remaining three variants, R160W, R184C and G204D showed considerably lower binding when compared to the WT (Figure 3A, lanes 6, 7 and 8, respectively). The quantification of the bound/unbound probe using a Phosphorimager showed a drastic decrease in binding of R184C to the probe, resulting in only 3.1% binding when compared to the WT, 100% binding (Figure 3B). The variants R160W and G204D resulted in 7.6% and 9.3% binding, respectively (Figure 3B). Even higher concentrations of these protein variants (40 μg) had comparable effect on RNA binding activity, and we still observed similar results (Figure S4). Additionally, to examine the strength of our selection criteria using these seven SNP prediction tools, we chose three SNP variants that were predicted not to affect the protein function (Figure 1A). These protein variants containing the following amino acid substitutions: D219E, T187S and S185N were also tested in EMSA. As shown in Figure S5, these nsSNPs did not affect RNA binding, confirming the accuracy of our multiple strategies. Finally, to test if all protein variants were similarly expressed, we performed Western blots using an anti-flag antibody and observed comparable expression (Figure 3C and Figure S6) when corrected by the endogenous beta-actin expression.

### Subcellular localization of nsSNPs-containing ZFP36L2 variants

The ZFP36 family members can shuttle from the nucleus to the cytoplasm upon mitogen stimulation [61]. These proteins contain a leucine-rich nuclear export signal (NES) that interacts with the nuclear export receptor CRM1/exportin 1 to actively move them to the cytoplasmic subcellular compartment [62]. Inhibition of CRM1 activity by agents like Leptomycin B resulted in nuclear accumulation of these proteins [62]. On the other hand, when only the tandem zinc finger domain is fused with GFP it leads to nuclear localization, suggesting that the tandem zinc finger domain contains a potential nuclear localization signal (NLS). However, the nuclear localization does not depend on the conformational integrity of zinc domains and does not require the functional RNA binding ability of this domain [62,63]. Because all our nsSNPs are in the tandem zinc finger domain, there was a chance that they could affect the subcellular localization. To investigate that possibility, we constructed green fluorescence protein fusions containing the WT-ZFP36L2 and its protein variants. The WT protein as well as variants were predominantly cytoplasmic when expressed in HEK 293 cells (Figure S7), suggesting that our nsSNPs do not affect the NLS and ability of these proteins to shuttle to the cytoplasm is preserved.

### Prediction of the effects of nsSNPs using Structure-based Analysis Tools

In some cases, a single amino acid alteration will have no effect on the overall protein folding. However, in other cases, it can completely alter the secondary and tertiary structures, and therefore also affect the biological activity. Given that structural changes may directly affect the protein’s function, we chose three bioinformatics tools, DUET, DynaMut and PyMOL, to analyse if the five nsSNPs identified by sequence-based approach would affect protein structure. Each of these prediction tools uses a different combination of datasets and parameters to generate a final algorithm; meaning that inevitably, the results and performances may differ. Therefore, we used multiple prediction programs to increase the likelihood of an accurate prediction.

DUET analysis is an integrated computational approach that combines results from two methods (mCSM and Site-Directed Mutator) in an optimized way using Support Vector Machines (SVMs) trained with Sequential Minimal Optimization. DUET predicted that all five mutants, Y154H, R160W, R184C, G204D, and C206F were in the category of mutations that decrease the protein stability, as shown in supplementary Table 5 and Figure S8. However, it is difficult to accurately interpret DUET results when the amino acid substitution results in only small ΔΔG differences [64]. proposed that only ΔΔG intervals bigger than ±0.5 kcal/mol should be considered significant using DUET. If this benchmark is used, only R184C and C206F would be considered significant using DUET, whereas the other variants would be comparable to the WT.

DynaMut predicted differences in all mutants when compared to the WT (supplementary Table 5 and Figure S9); however, these differences do not go in the same direction as DUET. In particular, the DynaMut prediction for C206F showed increased protein stability (supplementary Table 5), whereas DUET predicted decreased stability. In the case of C206F substitution that destroys the second zinc finger domain and results in a collapsed protein, the contradictory findings can be explained by the fact that this structural change is perceived differently by these two programs.

DynaMut results also include information about molecular flexibility using vibrational entropy energy (supplementary Table 5), whereas DUET does not. According to this, C206F led to less flexible protein structures (Figure S10). The structural conformation and flexibility of ZFP36L2 are expected to be crucial for its interactions with the RNA. Logically, it can exert both beneficial and detrimental effects on binding. It can be detrimental if specific amino acids proximal enough to hold a strong interaction with the RNA are no longer present in the novel predicted structure. Flexibility can be beneficial if it enables ZFP36L2 to accommodate RNA sequences, facilitating binding by allowing conformational adjustments that optimize the interaction. However, the significant decrease in protein flexibility as the case of C206F may just illustrate the disruption of the tandem zinc finger domain, known to abolish the protein/RNA interaction.

**Figure 4.**
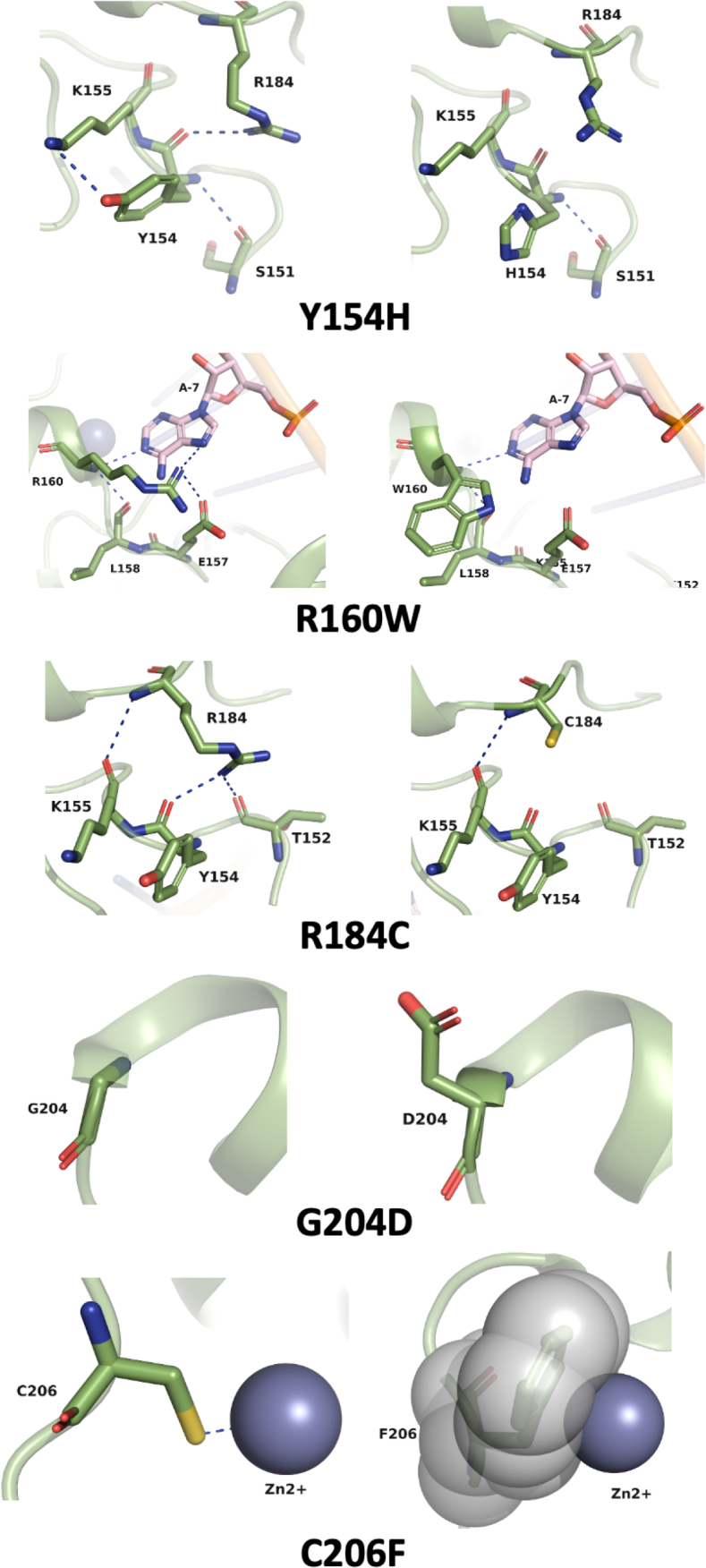
Molecular modeling of ZFP36L2 nsSNP variants in comparison with WT using PyMOL. On the left column is WT-ZFP36L2 and on the right are nsSNP variants. Each amino acid is represented by mint green sticks, with surrounding residues contributing to interactions. RNA nucleotides are depicted in pink, and Zn2+ is shown as a sphere. Hydrogen bonds are indicated by blue dotted lines. Additionally, the WT representation of the sidechain of C206F is used to visualize steric clashes with Zn2+.

Finally, we used the PyMOL mutagenesis tool to systematically explore the impact of each mutation with the surrounding amino acids, focusing on the hydrogen bond interactions. PyMOL predicted that four mutations Y154H, R160W, R184C, and C206F would have a deleterious structural impact, because they significantly disrupted crucial interactions within the protein structure, as shown in detail on Figure 4. Among these mutations, two variants, Y154H and R184C are notable for destroying hydrogen bonds with other proximal residues, K155/R184 and T152/Y154, respectively (Figure 4). R160W substitution disrupts interactions with critical adenine nucleotides in the RNA (Figure 4). Notably, the replacement of a bulky phenylalanine residue at the C206 position induces steric clashes between its side chain with the Zn^2+^ ion, further highlighting the expected structural consequences of this substitution resulting in the disruption of the second zinc finger domain. In contrast to the other variants, G204D substitution was interpreted by PyMol as a neutral effect, maintaining its solvent-exposed nature without significantly inducing any direct loss, apparently not affecting amino acid interactions. However, the substitution of a glycine to an aspartic acid would require more space to accommodate a larger amino acid that is also negatively charged.

In conclusion, when we compared DUET and DynaMut results we observed contradictory results: one predicts protein destabilization, whereas the other predicts stabilization. In some studies, DUET has been shown to perform better than DynaMut [64]; however, in the present study we do not know if the same conclusion is valid.

Using the AlphaFold 3.0 program, as illustrated in Figure S11A, B and C; we obtained comparable results as when we used PyMol as shown in Figure 4.

### Stability of protein variants using cellular temperature shift assay

**Figure 5.**
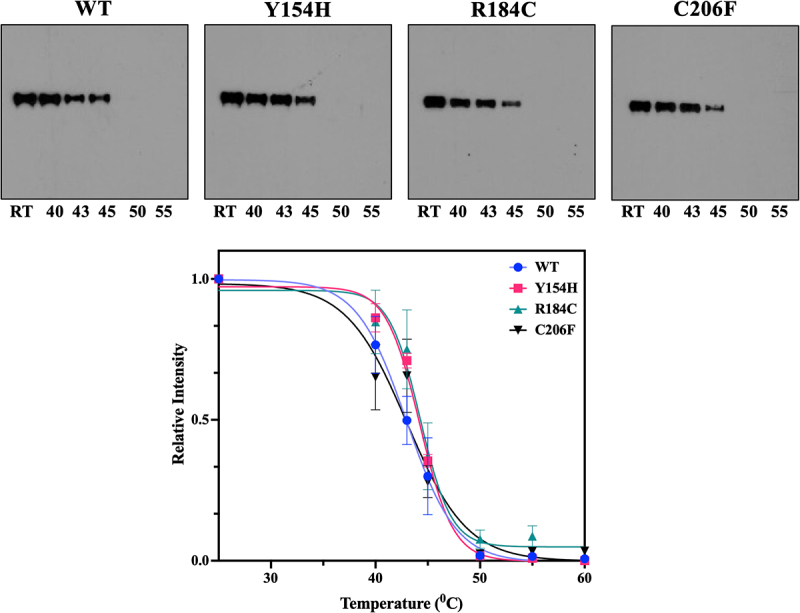
Cellular temperature shift assay of the WT ZFP36L2 and the tree protein variants that most affected binding, Y154H, R184C and C206F are comparable, suggesting similar protein stability.

The use of bioinformatic prediction programs to investigate the stability of these variant proteins such as DUET and DynaMut led us to conflicting results. DUET predicted that all five protein variants would have decreased protein stability, whereas DynaMut led to the result that some variants would be even more stable than the WT protein. Because these programs use different combinations of datasets and parameters to generate a final algorithm, it is not unexpected that the results and performances may differ. To address these contradictory results from the bioinformatic programs we considered possible ways to investigate the protein stability experimentally. Circular dichroism would have been ideal; however, it would require high concentrations of purified protein. We attempted to purify using a HEK cell overexpression system and Flag-Ab but unfortunately the concentration was not sufficiently high. As an alternative, we decided to perform a cellular temperature shift assay (CETSA). First, we determined the conditions of this assay using a broad range of temperatures, from 4°C to 75°C. Once we obtained this initial result, we tuned up the temperatures closer to the T_m_ of ZFP36L2 and performed three replicate experiments (Figure 5). In each of these independent replicates, all four proteins were tested on the same day to avoid biased variations. The result of these experiments suggests that the stability of the protein variants that most affect the RNA binding, Y154H, R184C and C206F, are comparable to the stability of the WT protein in CETSA assays. Thus, DUET and DynaMut structural prediction programs might not yet be sufficiently accurate in their prediction for this specific zinc finger domain.

### Molecular docking analysis

**Figure 6.**
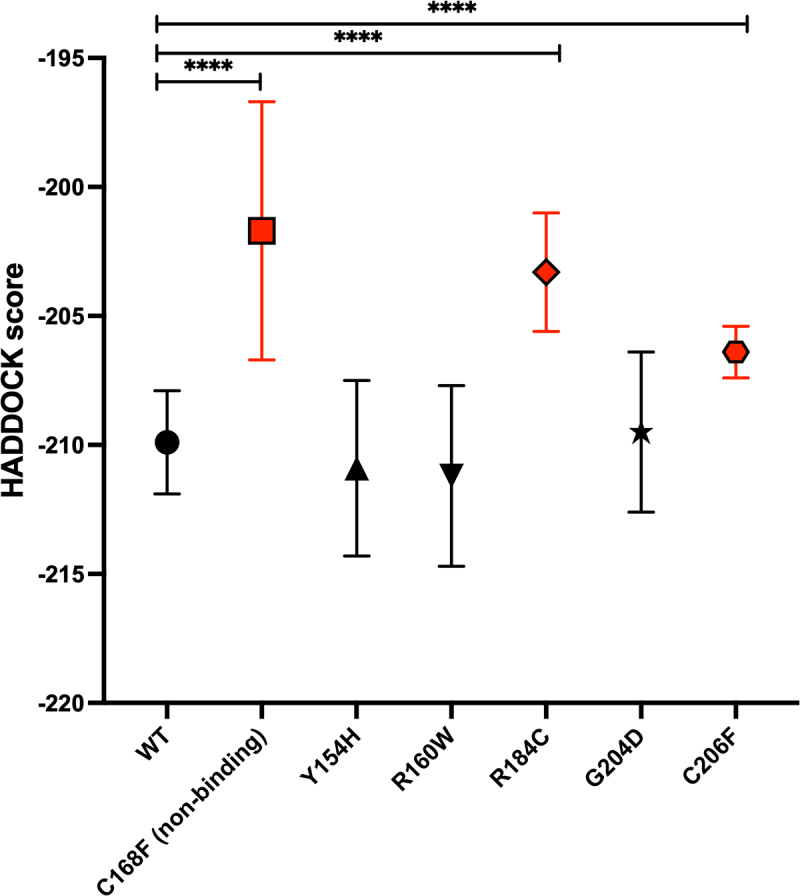
HADDOCK scores for the WT and each protein variant were obtained using the HADDOCK2.4 tool (supplementary table 6A). HADDOCK score was compared with the WT using one-way ANOVA, the number clusters for each interaction was considered as replicates, symbols in red illustrate statistically significant differences(****p < 0.0001).

Given that the ZFP36L2 protein does not trigger mRNA decay until it is bound to a particular ARE, understanding the structure of the tandem zinc finger domain variants bound to the ARE at an atomic level is crucial to gain insight into the molecular mechanisms and interaction performance. We used Molecular Docking to analyse this protein/RNA interaction. We performed the molecular docking of the WT and mutants to the 9-mer ARE (UUAUUUAUU) using HADDOCK2.4 and HDOCK tools. The final HADDOCK score is a function of a linear combination of various energies and buried surface areas. Higher HADDOCK scores (values towards zero) imply an unfavourable interaction, whereas lower scores, expressed by more negative values, are considered favourable. The highest HADDOCK score (value closer to zero) in comparison to the WT score was obtained with the mutant C168F (Figure 6), which is known to abolish the protein-RNA interaction. Additionally, this difference was statistically significant (*p* < 0.001), suggesting that our molecular docking assay was working as expected. The mutants R184C and C206F had higher HADDOCK scores than the WT and were both statistically significant (*p* < 0.001). These mutants displayed a behaviour comparable to the non-binding mutant control (C168F), suggesting an unfavourable interaction, which was also confirmed in our binding assays (Figure 3). The mutants Y154H, R160W and G204D displayed scores comparable to the WT (Figure 6 and supplementary Tables 6A and 6B). Interestingly, all three of these mutants displayed decreased binding in gel shift assays (Figure 3A); however, our molecular docking did not predict an overall or significant change in comparison to the WT (Figure 6). Individual amino acid changes on the interaction between each mutant protein and the 9-mer, such as hydrogen bonding and other close chemical interactions, identified in our docking analysis are displayed in Figure S12.

### Molecular dynamics simulations of the five validated nsSNPs

To gain deeper insights into the structural consequences contributing to the decreased binding observed in all five validated nsSNPs of ZFP36L2, we conducted molecular dynamics simulations to unravel the dynamic behaviour of biomolecular systems at an atomic level. In our study, we aimed to elucidate how these mutations affect the stability of protein-RNA complex by systematically quantifying H-bonds induced by the residue of interest with surrounding residues patterns throughout the molecular dynamic simulation trajectories. For that, we performed 1000 ns-long molecular dynamics simulations using the AMBER99SB force field and GROMACS 2020.3. We quantified the respective H-bond-forming capabilities with surrounding residues of the WT and mutant ZFP36L2 proteins. Throughout the course of the molecular dynamic simulations, we monitored the number of hydrogen bonds formed by residues of interest in both the WT and mutant ZFP36L2 proteins over time. We found notable differences in the hydrogen bonding patterns between the WT and mutant ZFP36L2 proteins. Specifically, we observed that the mutant-induced hydrogen bonds with distinct probabilities compared to the WT protein, indicating a potential disruption in the native hydrogen bonding network within the protein structure. For instance, mutations, such as Y154H and R160W, exhibited a higher probability of forming one or two hydrogen bond interactions with surrounding residues, respectively, when compared to their WT counterparts (Figure S13A and B). The R184C mutation abolished the number of hydrogen bonds formed with surrounding residues, decreasing from 6 to 3 as depicted in Figure S13C. This significant alteration in hydrogen bonding interactions suggests a structural perturbation induced by the mutation, potentially disrupting the stability and dynamics of the surrounding protein environment. Conversely, protein variants G204D and C206F displayed reduced probabilities of forming single hydrogen bonds with surrounding residues relative to the WT residues (Figure S13D and E). This divergence in H-bond probabilities highlights the distinct structural effects of the mutations on the surrounding residues, suggesting potential perturbations in the stability and dynamics of the ZFP36L2 protein.

## Discussion

ZFP36L2 belongs to the ZFP36 family of proteins, which function by binding to AU-rich elements preferentially located at the 3’-untranslated region (3’−UTR) of their specific target mRNAs. Upon binding, ZFP36L2 irreversibly accelerates the deadenylation of its bound RNA, the rate-limiting step in the degradation of most mRNAs. It is quite clear and widely accepted that the tandem zinc finger domain is the functional RNA-binding domain that characterizes this small family of proteins. In fact, 60% of the amino acids (48/79) that compose the tandem zinc finger domain are identical between the human orthologs ZFP36 (TTP), ZFP36L1 and ZFP36L2 (Figure S2). Interestingly, the sequence of amino acids in the tandem zinc finger domain of ZFP36L2 in human is the same as in mice. Given this highly conserved amino-acid sequence in the tandem zinc finger domain, it is understandable that all three proteins are capable of binding to the same iconic targets containing multiple AREs of TNF-alpha and GM-CSF mRNA in biochemical assays [22,26]. Intriguingly, individual knockout mouse of each of these proteins leads to different phenotypes, suggesting that many other factors influence the physiological roles of these proteins *in vivo*. ZFP36-KO leads to an inflammatory syndrome resembling lupus and rheumatoid arthritis [23,24]. ZFP36L1-KO is lethal due to chorioallantoide fusion defects [25]. ZFP36L2-KO leads to severe pancytopenia of all haematopoietic cell lines and in death at the second week of life [5,26].

To date, the only experimental structure obtained from this protein family is the structure of the tandem zinc finger domain of ZFP36L2 in complex with a 9-mer RNA (5’-UUAUUUAUU-3’) [50] (PDB ID: 1RGO) using NMR. The tandem zinc finger domain from the ZFP36 proteins is present in eukaryotic organisms distant from humans, including yeast and plants. Even in those distant species, the tandem zinc finger domain binds to target transcripts with precise affinity and evolutionary conserved functions [65]. Previous mutation studies of ZFP36 (TTP) [66], the prototype of this protein family, have shown that ‘although the majority of conserved residues within the tandem zinc finger domain of ZFP36 are required for productive binding, not all residues are functionally equivalent’. For example, they obtained results of at least two ZFP36 zinc finger mutants (E170R or E170V) which did not lead to negative effects on RNA binding. Based on this previous interpretation, we hypothesized that not all nsSNPSs in the tandem zinc finger domain of ZFP36L2 would have deleterious effect. Likely, a deleterious effect is somehow resultant of the location of the amino acid substitution as well as to how different the amino acids themselves are. Thus, in the present study, we aimed to identify nsSNPs located in the tandem zinc finger domain with potential deleterious effects on the RNA binding and consequently on the protein function. Initially, 514 nsSNPs located in the *ZFP36L2* gene were retrieved from NCBI dbSNP. Out of these 514 nsSNPs, 32 nsSNPs were in the functional zinc finger domain of ZFP36L2. We used multiple computational tools to better predict the possible effects of the nsSNPs located in the tandem zinc finger domain. We used seven different tools (PredictSNP, MAPP, PhD-SNP, PolyPhen-1, PolyPhen-2, SIFT, and SNAP) to predict nsSNPs with deleterious functional among these 32 SNPs. Using this strategy, we selected five nsSNPs, namely Y154H (rs375096815), R160W (rs1183688047), R184C (rs1214015428), G204D (rs1215671792), and C206F (rs920398592) predicted, by all seven tools, to have detrimental effects with functional repercussions. Alignment of different family members of different species (Figure S2) revealed that certain regions are highly conserved, such as the CCCH intervals within each finger and the lead-in sequences (YKTEL) to each finger, whereas other positions can potentially tolerate significant amino acid divergence. In terms of evolutionary conservation, we found that three among these five deleterious nsSNPs are positioned in amino acids with high conservation scores, whereas two presented moderate conservation scores (Figure 2). Therefore, we expected that at least three nsSNPs located in positions with high conservative scores: R184C, G204D, and C206F to have high probability of disrupting the binding affinity of ZFP36L2 to RNA.

We next used RNA gel shift assays (EMSA) to test the binding of each protein variant to a ^32^P labelled RNA probe. Using EMSA, we obtained consistent and reproducible results showing that all five nsSNPs predicted as potentially deleterious did indeed lead to reduced binding (Figure 3A). Conversely, three snSNPs predicted not to affect protein function presented normal RNA binding (Figure S5). These experimental results are in accordance with our predictions at the sequence level (Figure 1A). In fact, the only substitutions expected to ‘completely’ destroy binding in EMSA are the ones that coordinate the zinc ion (CCCH) and disrupt the first or the second zinc finger domain [50]. This is what we observed with C206F that disrupts the second zinc finger domain and is unable to bind to RNA (less than 1% in our EMSA). We were satisfied to see that Y154H and R184C, located at the leading sequence and linker, respectively, also completely abolish binding (<1% and 3.1%, respectively). These are local domains highly conserved and known to be important for the zinc finger domain [50]. The (R/K)YKTEL leading sequence has a role in backbone hydrogen-bonding interactions that is essential for the recognition of the U and A bases at the 5’-end of each half-site of the ARE. *Hudson et al*. determined that the Zinc Finger 1 (ZF1) and Zinc Finger 2 (ZF2) motifs constitute the two walls of a deep pocket that provides sites for the bases U6 and U2 in 9-mer, respectively. Additionally, it has been shown that substitutions of positively charged residues in the (R/K)YKTEL of both zinc fingers result in severe loss of RNA binding [66]. In our binding assay, Y154H led to one of the most dramatic decreases in binding (Figure 3B), comparable to the decrease of nsSNP C206F. This suggests that the Y154H substitution in the leading sequence can be functionally as important as the disruption of the tandem zinc finger domain itself, present in C206F. A substitution to histidine, a charged amino acid with an imidazole side chain, not only brings a positive charge but also requires more space to accommodate the imidazole chain (Figure 4), disrupting the interaction with U and A bases at the 5’-end of each half-site of the ARE and explaining the drastic effect decreasing the binding to RNA. When we tested R184C in terms of binding, we observed a significant impaired ability to bind to RNA, only 3.1% of the WT binding (Figure 3B). Thus, our structural folding predictions in combination with our experimental validation confirms the importance of a stable linker region that is somewhat rigid for the RNA binding to occur.

The protein variants R160W and G204D, resulted in 7.6% and 9.3% binding, respectively, when compared to the WT, 100% binding (Figure 3B). These last two variants reduce the binding by 10× in EMSA. Using PyMOL molecular modelling (Figure 4) we obtained explanations on how local structural changes lead to this decrease in binding. Combined with molecular dynamic simulations (Figure S12) we confirmed the disruption of relevant hydrogen bonds. In contrast, in our molecular docking (Figure 5), the HADDOCK scores of variants R160W, G204D and Y154H were not statistically different from the WT. In our specific case, the use of molecular docking to predict interaction was not as effective as our experimental results using EMSA. Our conclusion is that it remains challenging to predict RNA/protein interactions as they are often mediated by difficult to model bridging water molecules. Moreover, the negative charge of the RNA makes the estimation of the electrostatic component of the interaction particularly challenging to model. Nonetheless, our docking results did correctly predict a significant difference for two of the five nsSNPs: R184C and C206F. The poses suggest important conformational differences in the intermolecular interaction (Figure S12) consistent with the experimentally observed deleterious effect of these two protein variants. Thus, the molecular docking in our particular analysis has the sensitivity to detect binding disruption in the context of a major zinc finger domain disruption, such as in the variants R184C and C206F, where the zinc finger stacking is disrupted, or the zinc finger domain is collapsed, respectively. However, it may not be sensitive enough to detect other more subtle structure modifications.

Finally, protein stability is essential for the structural and functional activity of a given protein [67]. ZFP36L2 protein stability analysis using prediction programs such as DUET and DynaMut were contradictory and not helpful. Since these programs use different combinations of datasets and parameters to generate a final algorithm, it is not unexpected that the results and performances may differ. However, when we experimentally tested protein stability using CETSA, the protein variants with the most deleterious functional effect, Y154H, R184C and C206F, exhibited comparable stability as the WT protein in CETSA. In our case, structural stability prediction programs such as DUET and DynaMut were not sufficiently accurate, whereas experimental results using CETSA points to comparable protein stability when comparing these protein variants and the WT protein.

Our strategy to predict ‘pathogenicity’ of a nsSNP based on sequence was fully supported by our experimental results using EMSA. The structural modelling using PyMOL and AlphaFold3.0 offered details of how the amino acid substitutions can affect the interactions with other proximal amino acids in local space. However, protein stability prediction tools, such as DUET and DynaMut were not in accordance with our experimental data using cellular temperature shift assays, in which stability of protein variants exhibited comparable stability as the WT protein. Other computational tools, such as molecular docking and molecular dynamics simulations can be informative but still have limitations and do not always fully agree with experimental results.

Overall, our work shows that selected SNP variants observed in the human genome (from dbSNP) can have significant effects on protein/RNA interactions. It is important to note that all variants we tested have very small minor allele frequencies (between ≤0.001% to 0.007%) and are therefore exceedingly rare. Nonetheless, because they are so rare it is difficult to know if any of these SNPs would have functional repercussions *in vivo*. In conclusion, our work shows that it is possible to selectively predict and test SNPs that disrupt RNA/protein interactions. As far as we know, none of the five deleterious nsSNPs in the *ZFP36L2* gene described here have been associated with any disease phenotype, likely because they occur with extremely low frequency in the general population. Nonetheless, our study offers a novel approach on how amino acid changes in specific positions, which are not directly involved in the protein/RNA interaction, can still have an impact on the binding and likely regulate the expression of variety of genes.

## Supplementary Material

9_Supplementary_Tables_Ressub.pdf

8_Supplementary Figures_Ressub.pdf

## Data Availability

All the SNPs used in this paper are publicly available in the NCBI dbSNP data repository. The selected 32 nsSNPs located in the tandem zinc finger domain of ZFP36L2 are listed in supplementary Tables 1, 2 and 4. The novel molecular structural models of all five deleterious nsSNPs created using computational modelling are included in the supplementary Figures S8, S9 and S10, as well as the visualization of the molecular docking in supplementary Figure S11.
