# Supplementary material for: Identification of deleterious non-synonymous single nucleotide polymorphisms in the mRNA decay activator ZFP36L2
Source: RNA Biol. 2024 Dec 13;22(1):1–15. doi: 10.1080/15476286.2024.2437590 (PMC12710939; doi:10.1080/15476286.2024.2437590)
Supplement: 9_Supplementary_Tables_Ressub.pdf [file KRNB_A_2437590_SM5252.pdf]

Supplementary Table 1. Expected accuracies in % according to PredictSNP-tools result. Deleterious variations are highlighted red and neutral ones are dark green in PredictSNP. Whereas in other tools, deleterious and neutral variations represented as pink and light green, respectively.

	**Mutation**	**PredictSNP**	**MAPP**	**PhD-SNP**	**PolyPhen-1**	**PolyPhen-2**	**SIFT**	**SNAP**
rs759036795	S151A	61%	82%	55%	67%	43%	53%	56%
rs1553341999	T152P	51%	66%	73%	67%	69%	79%	50%
**rs375096815**	**Y154H**	**87%**	**78%**	**88%**	**59%**	**65%**	**79%**	**72%**
rs1437668911	R160L	76%	84%	88%	67%	56%	79%	81%
**rs1183688047**	**R160W**	**87%**	**77%**	**82%**	**74%**	**65%**	**79%**	**81%**
rs770064767	P161A	60%	59%	72%	67%	61%	53%	72%
rs1469365465	P161L	55%	63%	68%	67%	43%	79%	62%
rs1261170219	S165G	65%	74%	72%	67%	63%	46%	56%
rs771017073	S165N	74%	74%	83%	67%	68%	90%	62%
rs1280967612	S165R	72%	82%	58%	67%	45%	46%	72%
rs746925321	G166R	76%	88%	88%	67%	65%	79%	89%
rs754529710	K169T	72%	84%	73%	67%	50%	53%	72%
rs1387415553	G171A	72%	77%	86%	67%	59%	46%	85%
rs1244959112	H181Y	61%	65%	73%	67%	47%	79%	62%
**rs1214015428**	**R184C**	**87%**	**91%**	**88%**	**74%**	**68%**	**79%**	**72%**
rs759784530	S185N	83%	75%	72%	67%	63%	70%	50%
rs1237026011	L186V	75%	76%	78%	67%	61%	66%	55%

rs1274850041	T187S	83%	80%	78%	67%	71%	78%	55%
rs773191930	R188L	72%	84%	89%	67%	43%	79%	72%
rs758876800	H189P	72%	88%	88%	67%	40%	53%	72%
rs1399663689	P190Q	76%	86%	86%	67%	59%	79%	62%
rs1174004908	K191R	55%	56%	61%	67%	43%	43%	55%
rs1422966999	Y192F	72%	77%	73%	67%	50%	45%	62%
rs1487768884	T199S	61%	76%	51%	67%	41%	45%	62%
rs8098	I203M	61%	62%	68%	67%	59%	79%	56%
**rs1215671792**	**G204D**	**87%**	**88%**	**88%**	**59%**	**63%**	**53%**	**89%**
**rs920398592**	**C206F**	**87%**	**77%**	**89%**	**74%**	**81%**	**79%**	**89%**
rs1315087781	P207L	72%	59%	77%	67%	47%	79%	62%
rs1356301633	P207S	60%	62%	45%	67%	47%	53%	58%
rs760808563	N217D	55%	59%	55%	67%	40%	46%	56%
rs1477249578	N217K	72%	57%	82%	67%	54%	53%	81%
rs201694029	D219E	83%	77%	72%	67%	64%	87%	67%

Supplementary Table 2. Classification of variants into disease-related or neutral missense variants by using GO annotations **by** SNPs&GO. A probability score > 0.5 indicates a disease-related mutation.

**Mutation**	**Prediction**	**Probability**
S151A	Neutral	0.222
T152P	Neutral	0.460
**Y154H**	**Disease**	**0.505**
R160L	Neutral	0.359
R160W	Neutral	0.438
P161A	Neutral	0.044
P161L	Neutral	0.127
S165G	Neutral	0.036
S165N	Neutral	0.013
S165R	Neutral	0.060
G166R	Neutral	0.277
K169T	Neutral	0.161
G171A	Neutral	0.401
H181Y	Neutral	0.462
**R184C**	**Disease**	**0.666**
S185N	Neutral	0.101
L186V	Neutral	0.096
T187S	Neutral	0.041
**R188L**	**Disease**	**0.694**
**H189P**	**Disease**	**0.653**
**P190Q**	**Disease**	**0.669**
K191R	Neutral	0.126
Y192F	Neutral	0.467
T199S	Neutral	0.237
I203M	Neutral	0.458
**G204D**	**Disease**	**0.785**
**C206F**	**Disease**	**0.938**
**P207L**	**Disease**	**0.775**
**P207S**	**Disease**	**0.646**
N217D	Neutral	0.273
N217K	Neutral	0.323
D219E	Neutral	0.043

Supplementary Table 3. Sequence-based prediction of the functional consequences of amino-acid substitutions in proteins by MutationAssessor. Variants with a FI score > 2.00 are predicted as deleterious variants.

Mutation	FI Score	Functional Impact (FI)
I203M	2.445	Medium
D219E	425	Neutral
Y154H	2.785	Medium
G166R	2.73	Medium
K169T	1.38	Low
H189P	2.255	Medium
S151A	1.955	Medium
S185N	0.48	Neutral
N217D	1.91	Low
P161A	0.74	Neutral
S165N	0.3	Neutral
R188L	2.78	Medium
**C206F**	**4.71**	**High**
K191R	1.785	Low
R160W	2.525	Medium
**R184C**	**4.47**	**High**
**G204D**	**3.92**	**High**
L186V	0.53	Neutral
H181Y	2.64	Medium
S165G	925	Low
T187S	-0,79	Neutral
S165R	0.98	Low
P207L	3.12	Medium
P207S	2.595	Medium
G171A	2.21	Medium
P190Q	2.91	Medium
Y192F	2.41	Medium
R160L	2.07	Medium
P161L	965	Low
N217K	2.585	Medium
T199S	1.345	Low
T152P	1.22	Low

Supplementary Table 4. Results of sequence-based analysis conducted with PredictSNP, SNPs&GO and MutationAssessor.

Mutation	Y154H (M1)	R160W (M2)	R184C (M3)	G204D (M4)	C206F (M5)
rsID	rs375096815	rs1183688047	rs1214015428	rs1215671792	rs920398592
PredictSNP: Expected Accuracies (in %)					
**PredictSNP**	**87%**	**87%**	**87%**	**87%**	**87%**
MAPP	78%	77%	91%	88%	77%
**PhD-SNP**	**88%**	**82%**	**88%**	**88%**	**89%**
PolyPhen-1	59%	74%	74%	59%	74%
PolyPhen-2	65%	65%	68%	63%	81%
SIFT	79%	79%	79%	53%	79%
SNAP	72%	81%	72%	89%	89%
SNPs&GO: Probabilities (Disease-related Variation > 0.5)					
**SNPs&GO**	**0.505**	0.438	**0.666**	**0.785**	**0.938**
MutationAssessor: Functional Impact Scores (High: FI > 3.5 / Medium: 1.9 < FI ≤ 3.5 / Low: 0.8 < FI ≤ 1.9 / Neutral: FI ≤ 0.8)					
MutationAssessor	2.785	2.525	4.47	3.92	4.71

Supplementary Table 5. Effects of nsSNPs on protein stability and molecular flexibility from DUET and DynaMut server.

Mutation	DUET	DynaMut	
	ΔΔG DUET [kcal/mol]	ΔΔG DynaMut [kcal/mol]	$\Delta\Delta S_{Vib}$ ENCoM [$kcal \cdot mol^{-1} \cdot K^{-1}$)]
Y154H (M1)	– 0.226 (↓S)	0.013 (↑S)	0.109 (↑F)
R160W (M2)	– 0.099 (↓S)	0.294 (↑S)	0.023 (↑F)
R184C (M3)	– 0.700 (↓S)	– 1.341 (↓S)	0.260 (↑F)
G204D (M4)	– 0.108 (↓S)	– 0.519 (↓S)	0.022 (↑F)
C206F (M5)	– 1.786 (↓S)	0.748 (↑S)	– 0.844 (↓F)

(↓S): Decreased Protein Stability | (↑S): Increased Protein Stability
(↓F): Decreased Molecular Flexibility | (↑F): Increased Molecular Flexibility

Supplementary Table 6A. Molecular docking results of ZFP36L2 and 9Mer RNA performed by HADDOCK2.4.

Protein-RNA Complexes	HADDOCK Score	Cluster Size	RMSD from the Overall Lowest-Energy Structure	Z SCORE	Buried Surface Area	Van der Waals energy	Electrostatic energy	Desolvation energy	Restraints violation energy
**Wild-Type-9MER**	**– 209.9 ± 2.0**	**62**	**1.0 ± 0.2**	**– 1.8**	**2414.9 ± 20.8**	-127.5 +/- 3.2	-357.3 +/- 13.9	-12.7 +/- 2.8	17.5 +/- 14.1
**Y154H (M1)-9MER**	– 210.9 ± 3.4	25	0.7 ± 0.4	– 2.1	2395.7 ± 25.0	-128.8 +/- 2.1	-356.8 +/- 19.5	-13.2 +/- 1.7	24.3 +/- 12.1
**R160W (M2)-9MER**	– 211.2 ± 3.5	59	0.6 ± 0.4	– 2.0	2427.7 ± 21.4	-132.9 +/- 2.5	-336.6 +/- 31.1	-12.8 +/- 1.4	18.4 +/- 11.7
**C168F-9MER***	– 201.7 ± 5.0	65	0.6 ± 0.3	– 2.2	2370.2 ± 43.8	-124.3 +/- 2.2	-342.9 +/- 28.7	-12.3 +/- 0.9	34.2 +/- 2.5
**R184C (M3)-9MER**	– 203.3 ± 2.3	56	1.0 ± 0.2	– 1.8	2333.5 ± 49.0	-125.5 +/- 3.1	-338.7 +/- 15.2	-12.0 +/- 1.2	18.8 +/- 15.1
**G204D (M4)-9MER**	– 209.5 ± 3.1	59	0.6 ± 0.4	– 2.2	2416.3 ± 35.1	-129.4 +/- 4.5	-349.3 +/- 11.8	-12.1 +/- 1.1	19.1 +/- 15.6
**C206F (M5)-9MER**	– 206.4 ± 1.0	74	0.7 ± 0.4	– 2.0	2397.9 ± 10.4	-123.5 +/- 3.2	-358.0 +/- 20.4	-13.2 +/- 2.8	17.8 +/- 12.9

*** C168F, a known disrupting mutation, was used as a control.**

Supplementary Table 6B. Molecular docking results of ZFP36L2 and 9Mer RNA performed by HDOCK.

	**WT**	**Y154H (M1)**	**R160W (M2)**	**R184C (M3)**	**G204D (M4)**	**C206F (M5)**
**Rank**	**1**	**1**	**1**	**1**	**1**	**1**
**Docking Score**	-794.34	-782.83	-798.14	-795.95	-794.15	-799.99
**Confidence Score**	10.000	10.000	10.000	10.000	10.000	10.000
**Ligand rmsd (Å)**	0.20	0.35	0.19	0.20	0.23	0.22
