# Supplementary material for: Identification of deleterious non-synonymous single nucleotide polymorphisms in the mRNA decay activator ZFP36L2
Source: RNA Biol. 2024 Dec 13;22(1):1–15. doi: 10.1080/15476286.2024.2437590 (PMC12710939; doi:10.1080/15476286.2024.2437590)
Supplement: 8_Supplementary Figures_Ressub.pdf [file KRNB_A_2437590_SM5251.pdf]

Supplementary Figures

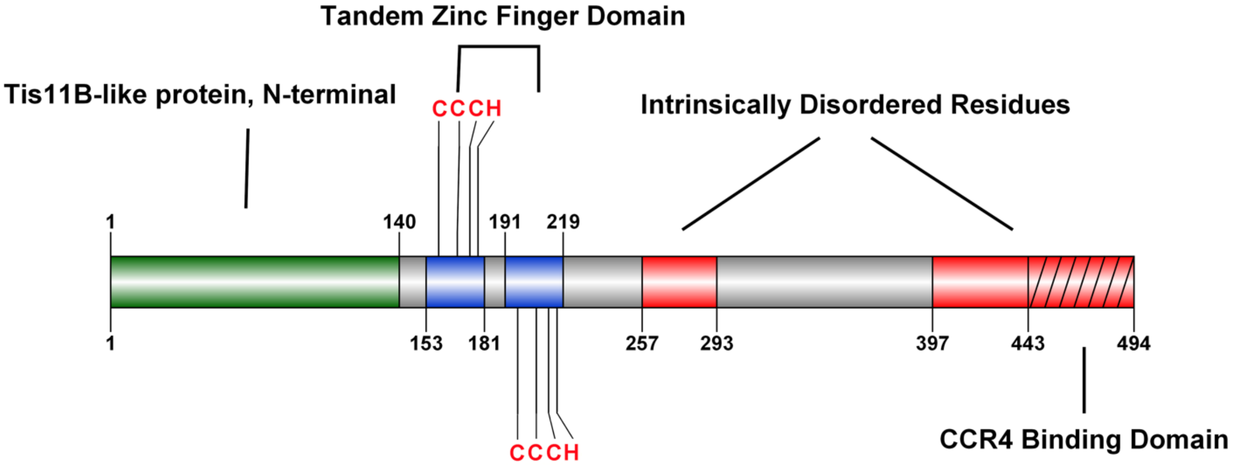

Figure S1. Schematic diagram of human ZFP36L2 protein domains using DOG 2.0 tool (Ren et al. 2009). The protein is composed of 494 amino acids. The two predominant domains are the Tis11B-like protein, N-terminal (1-140 residues) (InterPro ID: IPR007635/in green) and the Tandem Zinc finger (TZF) domain of the CCCH-type (InterPro ID: PR000571/ in blue). Each of the zinc finger domain contains three cysteines and one histidine residues represented as C and H that coordinate the zinc ion. Their specific locations are Cys159, Cys168, Cys174 and His178 residues (CCCH) and Cys197, Cys206, Cys212 and His216 residues (CCCH) in the first and second domains, respectively. Additionally, 257-293 and 397-494 are predicted as intrinsically disordered residues by MobiDB tool (in red). Hatching region between residues 443-494 is defined as the CCR4 binding domain (Zheng et al. 2022).

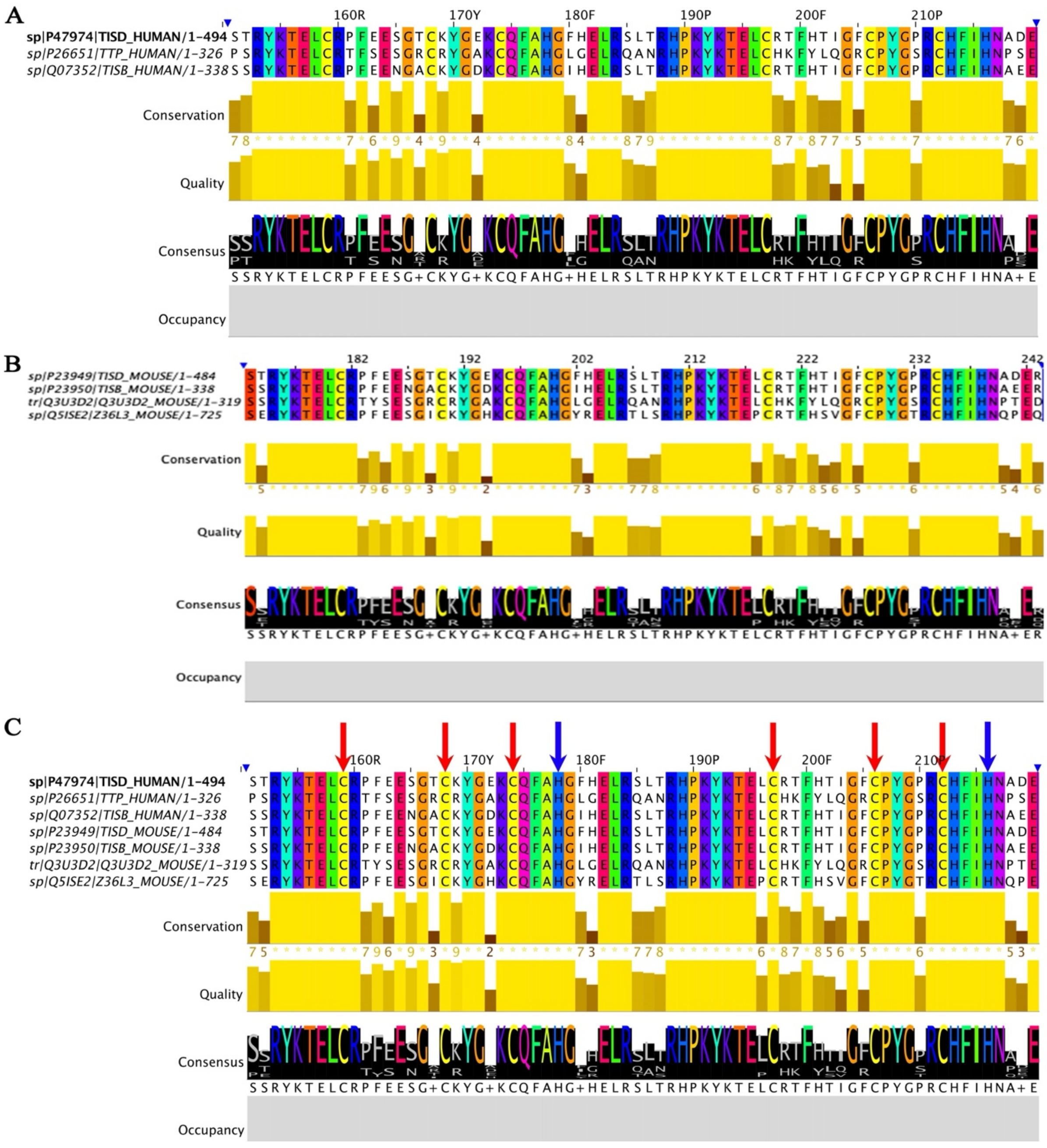

Figure S2. Sequence alignment of ZFP36 (TTP) family members using Jalview (Clustal OWS Alignment). (A) Alignment of all three human ZFP36 family members: TISD_HUMAN (P47974), TTP_HUMAN (P26651), and TISB_HUMAN(Q07352). (B) Alignment of all four mouse ZFP36 (TTP) family members: TISD_MOUSE (P23949), TISB_MOUSE (P23950), Q3U3D2_MOUSE (Q3U3D2), and Z36L3_MOUSE (Q5ISE2). (C) Alignment of all human and mouse ZFP36 family members. CCCH-motifs are indicated by successive three red arrows and one blue arrow.

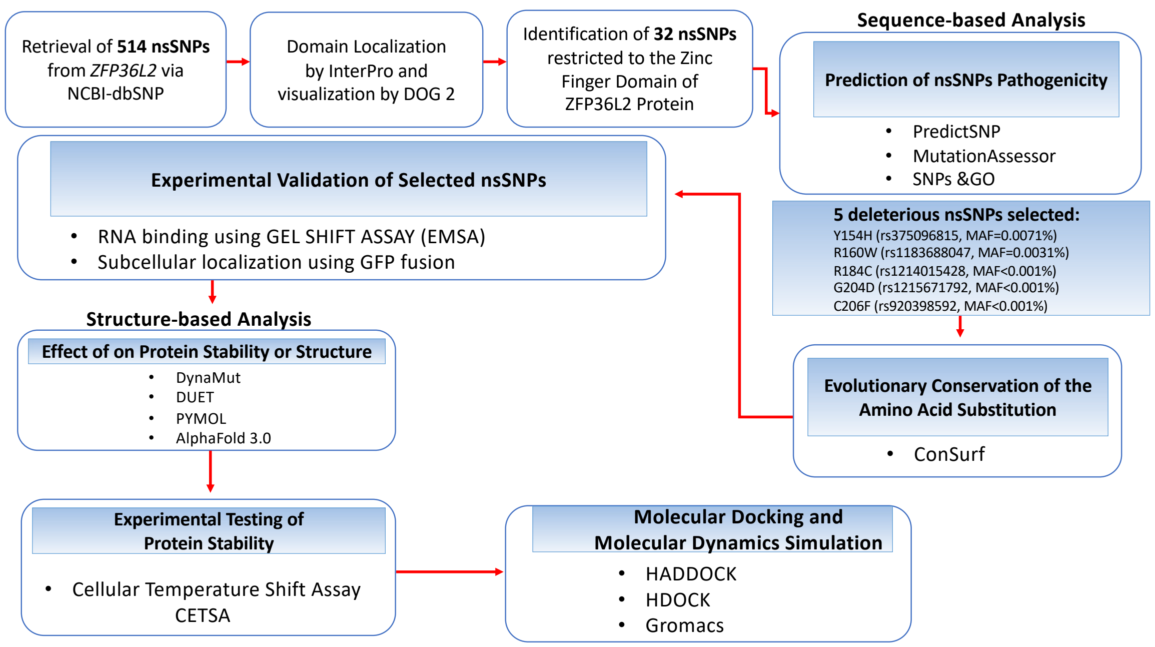

Figure S3. Methodological workflow of the study.

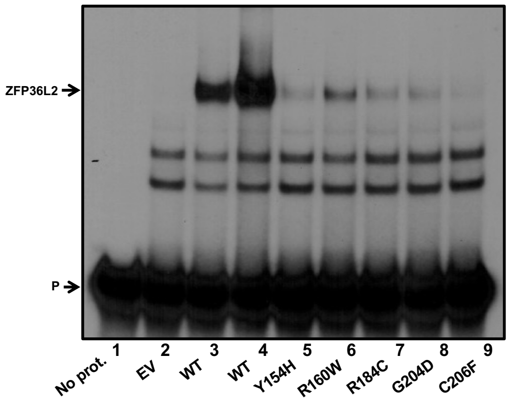

Figure S4. ZFP36L2 protein variants expressing the five nsSNPs predicted to be deleterious were compared to the WT protein ability to bind to RNA. RNA electrophoretic mobility shift assays were performed by incubating 0.2×10^5 cpm of *Gm-csf* probe with protein extracts from HEK 293 cells transfected with empty vector (EV, lane 2) or with a vector expressing the WT (lane 3 and 4 contains 20μg and 40 μg of protein, respectively) or different ZFP36L2 variants (in lanes 5 to 9, each lane containing 40μg of protein extracts, double the amount used in Figure 3A). Lanes 5 to 9 correspond to Y154H, R160W, R184C, G204D and and C206F, respectively.

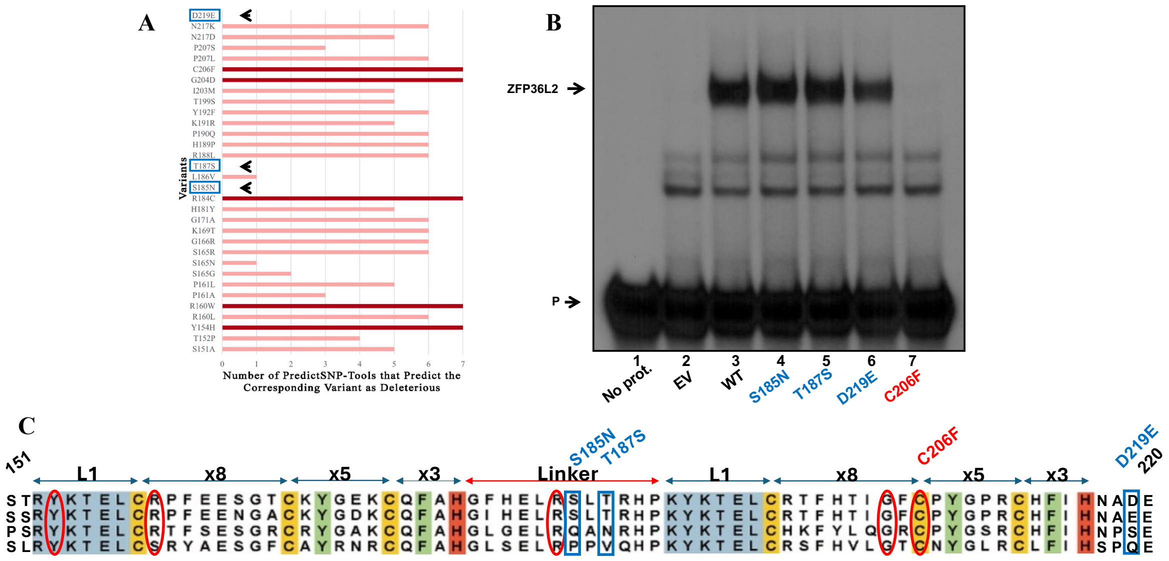

Figure S5. ZFP36L2 protein variants expressing the three nsSNPs predicted to be non-deleterious were compared to the WT protein ability to bind to RNA. (A) List of 32 SNP variants identified by PredictSNP-tools (PredictSNP, MAPP, PhD-SNP, PolyPhen-1, PolyPhen-2, SIFT, and SNAP). Three variants were simultaneously classified as functionally non-deleterious by all 7 classifiers (arrows). (B) RNA electrophoretic mobility shift assays were performed by incubating 20 µg of protein extracts from HEK 293 cells transfected with empty vector (EV, lane 2) or with a vector expressing the WT or different ZFP36L2 variants (lanes 3 to 7) with 0.2×10^5 cpm of *Gm-csf* probe. Lane 7 corresponds to a protein variant shown in Figure 3A, where the second zinc finger domain was disrupted abrogating binding (C206F). Lanes 3 to 6 correspond to S185N, T187S and D219E, respectively. Alignment of tandem zinc finger of hZFP36L2 (TIS11D), hZFP36L1 (TIS11B), hZFP36 (TTP), and Xenopus. The location of each three non-deleterious nsSNPs is illustrated in blue.

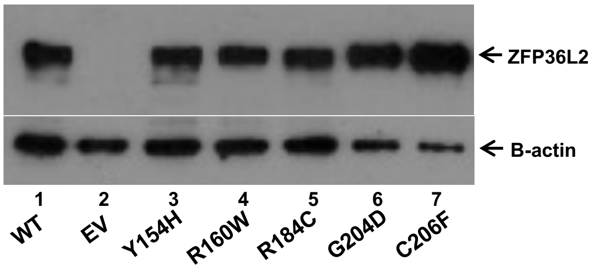

Figure S6. Immunoblot using less protein for a shorter time exposure. Two and a half μg of protein extracts (half of the amount shown in figure 3C) were loaded per lane and probed with a Flag antibody (top panel) or beta-actin (lower panel). The membrane was exposed to film for 3 seconds.

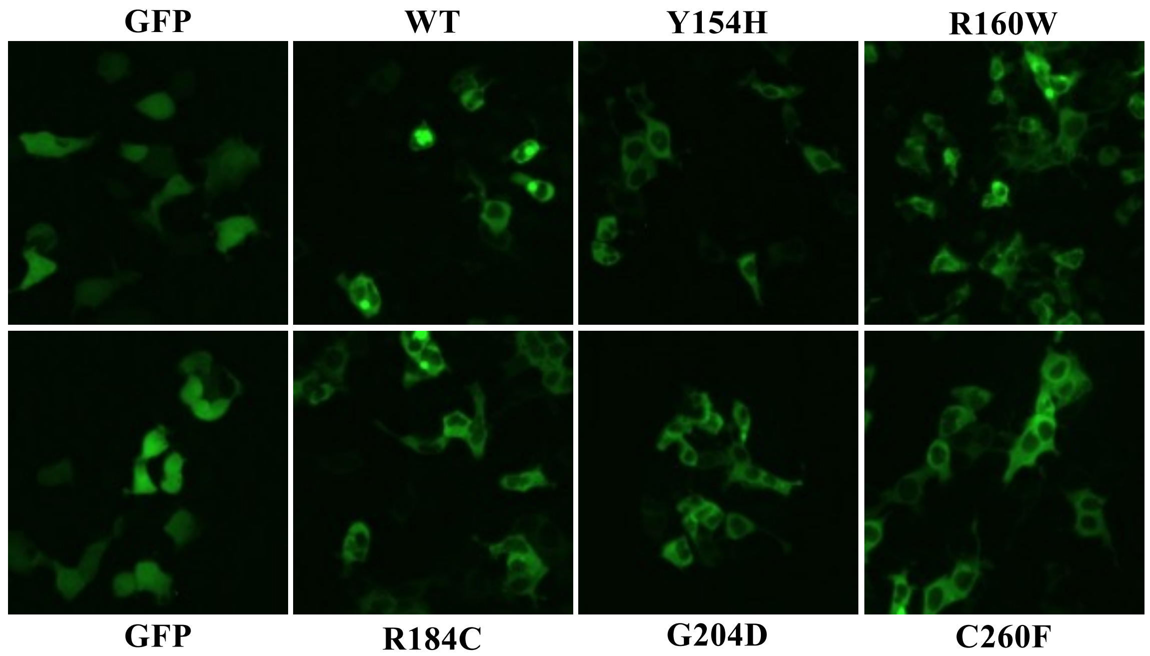

Figure S7. HEK 293 cells were transiently transfected with 500 ng of GFP or each GFP-ZFP36L2 constructs expressing protein variants or the WT protein. Cells were visualized with a Nikon ECLIPSE Ti2 inverted microscope with a spinning disk for live cell imaging at 10 x.

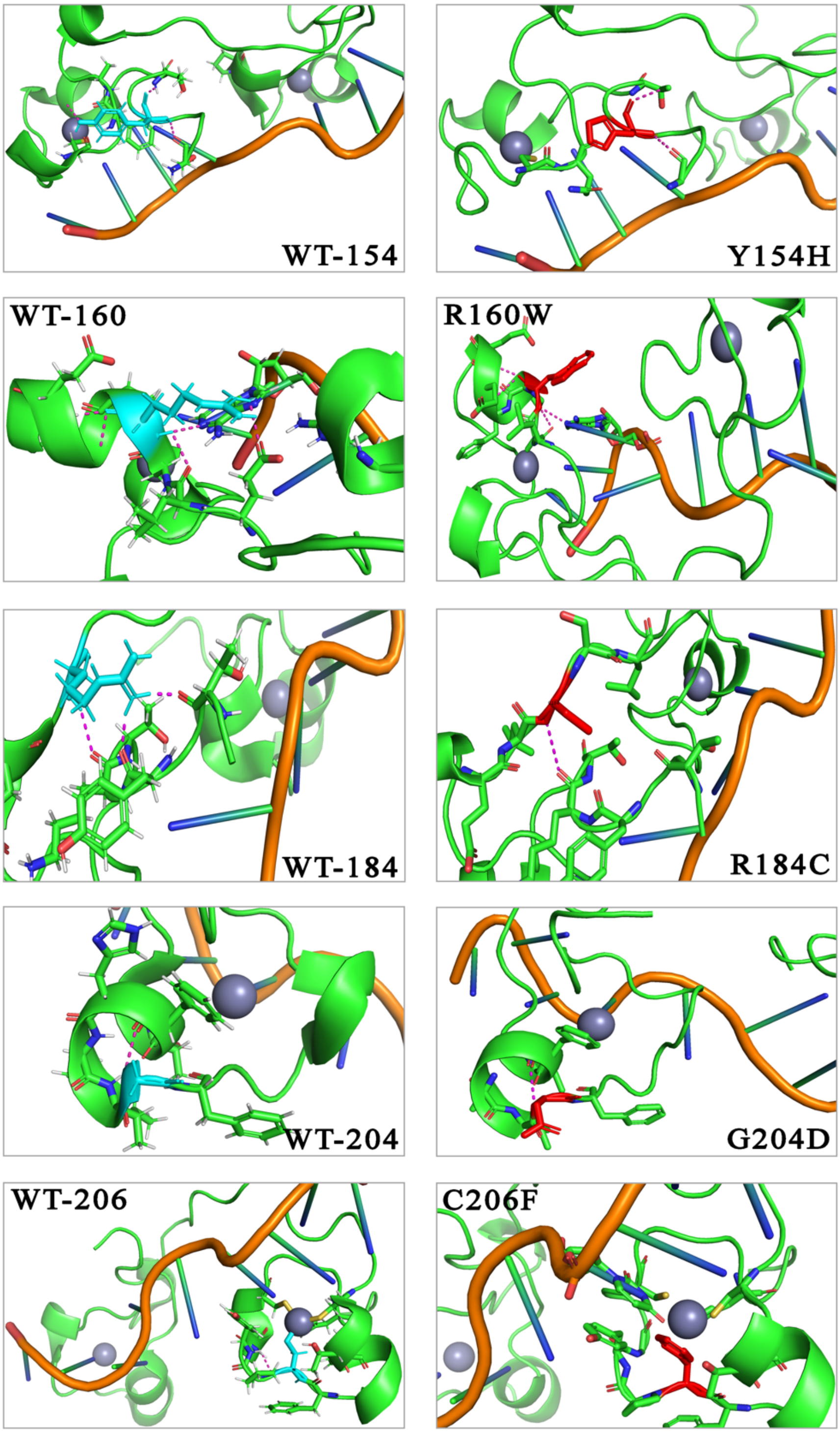

Figure S8. Visualization of interatomic interactions of ZFP36L2 wild-type and mutants predicted by DUET using Pymol. Right column illustrates WT residues in cyan and mutant residues on the right column are in red, pink dotted lines indicate hydrogen bonding between surrounding of target residues.

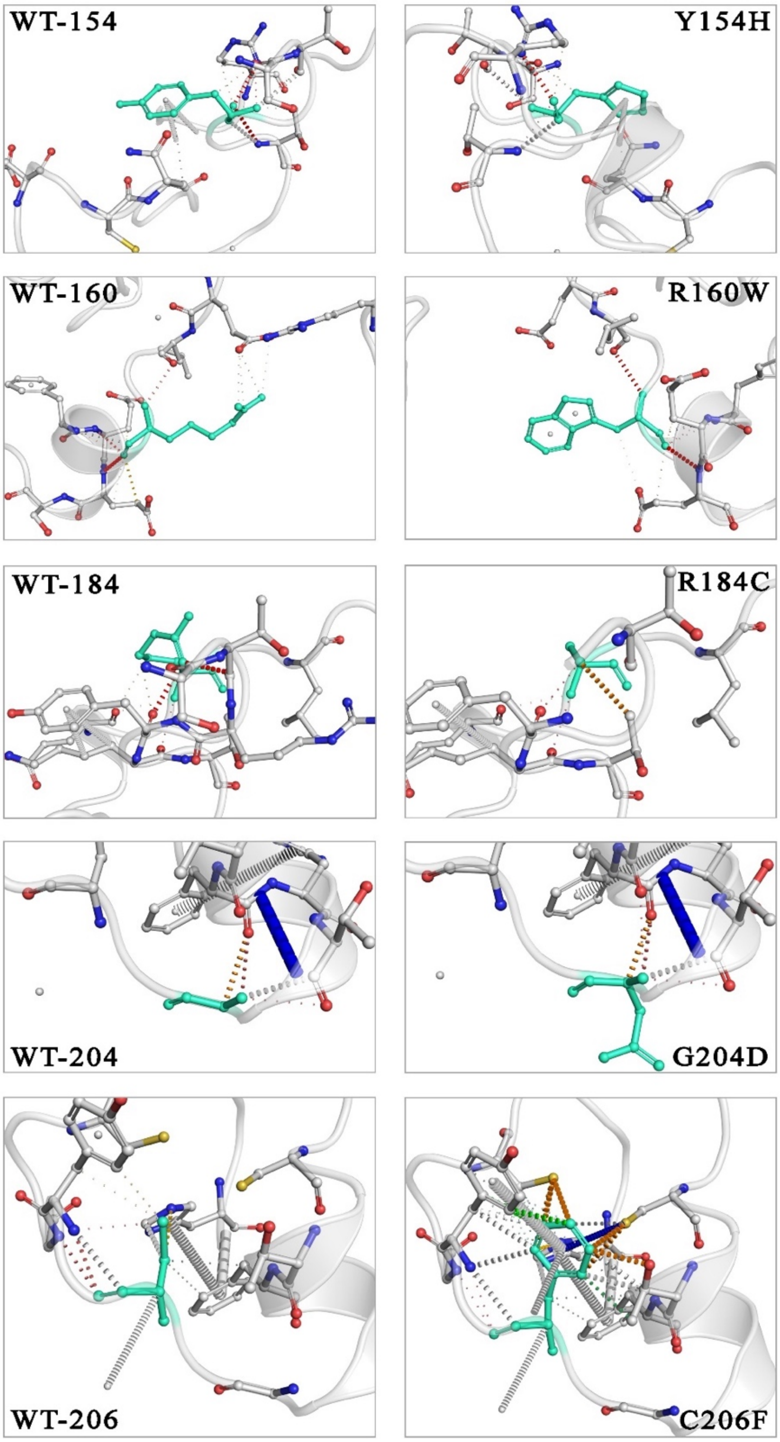

Figure S9. DynaMut: Predicted interatomic interactions of ZFP36L2 wild-type and protein variants. The corresponding amino acid substitutions are shown as mint green sticks along with the surrounding residues, which in turn participate in interactions. Aromatic interactions are shown in light blue, carbonyl interactions in pink, halogen bonds in blue, weak hydrogen bonds are shown in orange, hydrogen bonds in red, hydrophobic contacts in green, ionic interactions in yellow, van der Waals interactions in grey. Bonds and interactions are indicated with dashed lines.

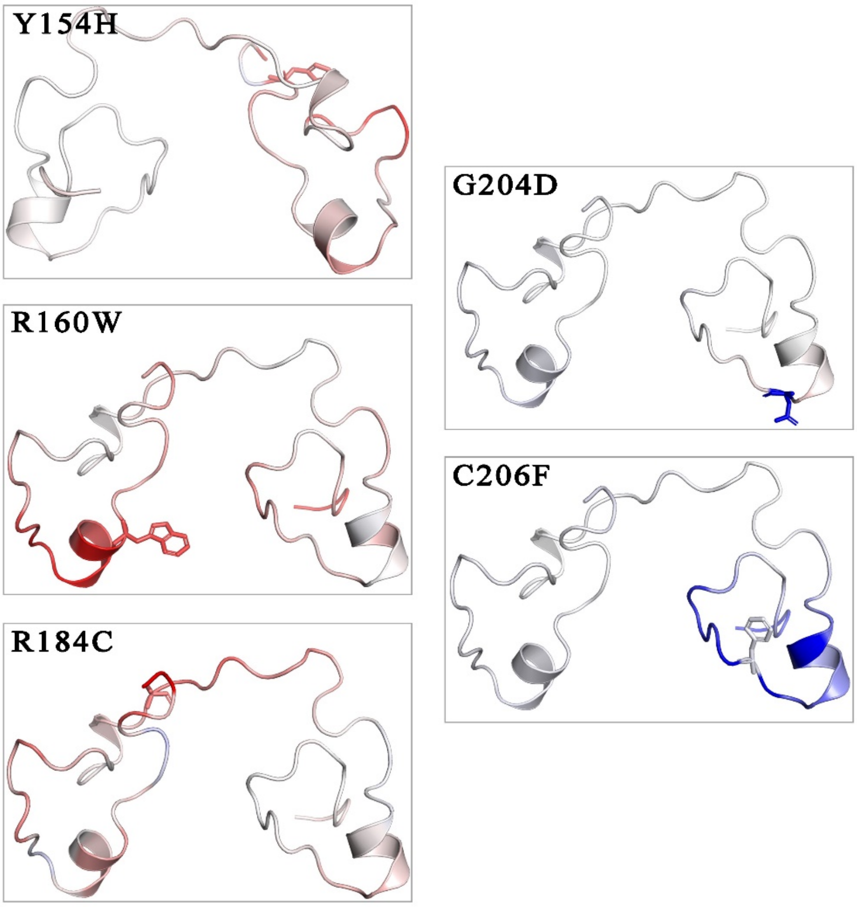

Figure S10. Vibrational entropy energies of target variations differ from those of the ZFP36L2 wild-type protein by DynaMut. Red color indicates a gain in molecular flexibility, and blue a rigidification of the structure.

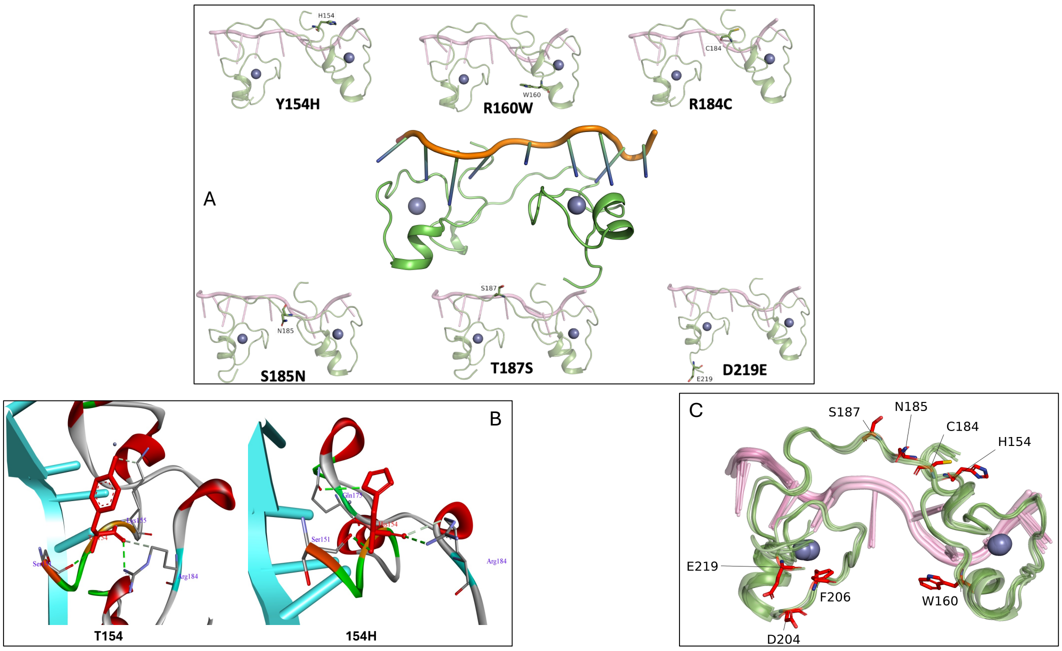

Figure S11. Conformation of the WT zinc finger domain and protein variants using AlphaFold predictions. (A) In the central part is illustrated the WT zinc finger domain (green), the zinc ions (blue spheres), and the 9-mer-RNA (UUAUUUAUU) in orange and bluish bars. Above the WT, are three protein variants deleterious and below are the three non-deleterious. The protein variants are colored in light green and the RNA in light pink. (B) Zoom view of how the tyrosine on position 154 directly interact with the RNA, here represented in blue. Once this tyrosine is substituted to a histidine, the local hydrogen bonds and other adjacent interactions are altered, making virtually impossible to accommodate the RNA. (C) In green is the superimposed conformation of all protein variants investigated here.

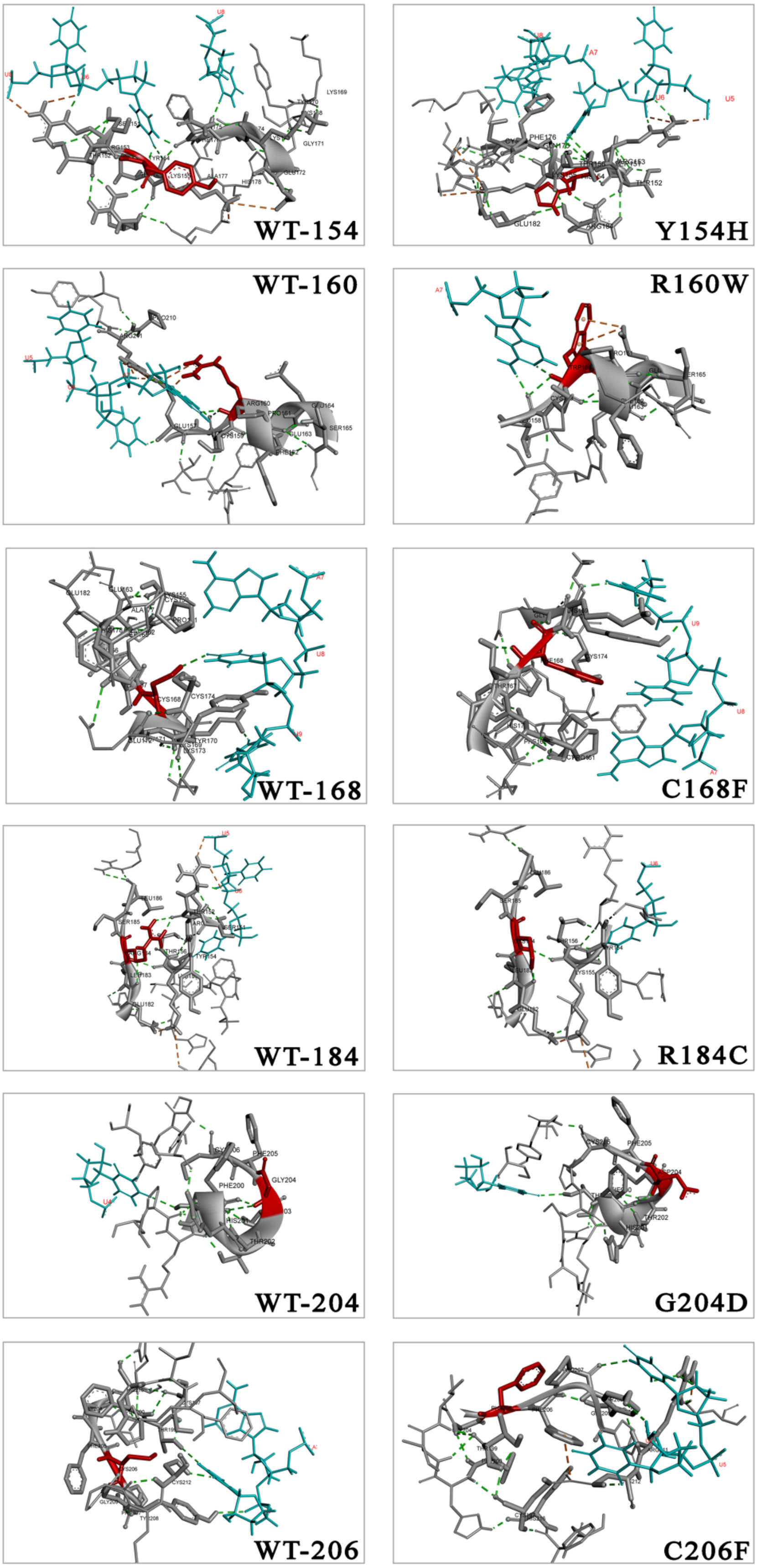
WT-154
Y154H
WT-160
R160W
WT-168
C168F
WT-184
R184C
WT-204
G204D
WT-206
C206F

Figure S12. Visualization of docking poses by HADDOCK2.4 of TZF domain of ZFP36L2 mutants and RNA oligonucleotide using BIOVIA Discovery Studio 2021. Red and cyan colors indicate the corresponding mutated residue and RNA oligonucleotide, respectively. Green and orange/brown color indicate the hydrogen bonding and the electrostatic interactions of selected mutated residue to its surrounding, respectively. Residues in 5Å to the mutated residue are shown as stick representation.

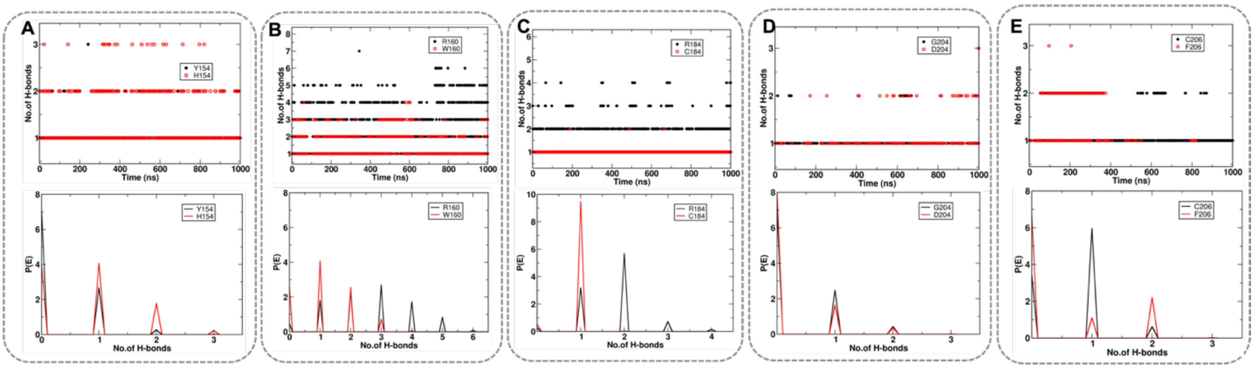

Figure S13. Comparative analysis of hydrogen bonding patterns in WT and mutants**.** Temporal evolution of H-bond presence with respect to time (ns) is depicted for WT (A) and mutant ZFP36L2 proteins (B-E; Y154H, R160W, R180C, C206F, and G204D, respectively). Briefly, we evaluated the formation of H-bonds between residues of interest and surrounding residues throughout the simulation trajectory. For each variant, the molecular simulation was performed three times. Respective probability distributions of H-bonds are presented below each plot, offering insight into the differences in H-bond-forming capabilities between WT and mutant variants.

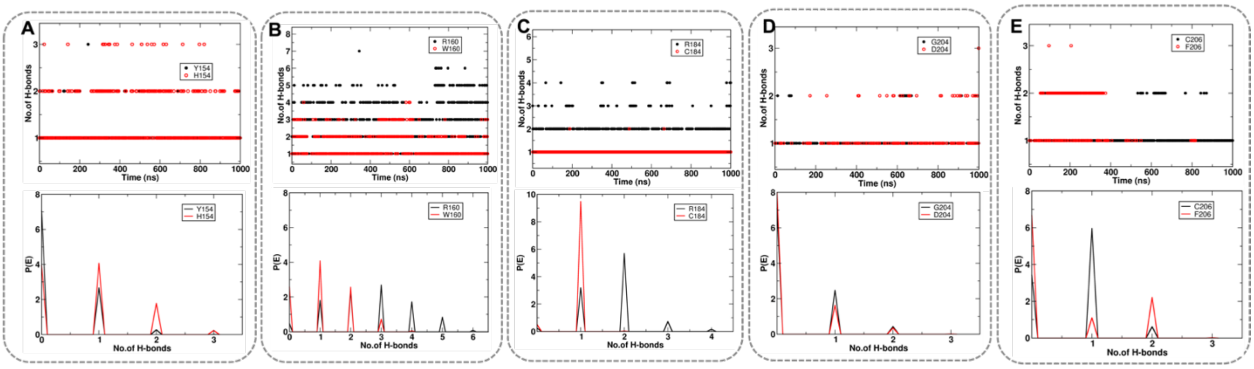

Figure S13. Comparative analysis of hydrogen bonding patterns in WT and mutants**.** Temporal evolution of H-bond presence with respect to time (ns) is depicted for WT. (A) and variant ZFP36L2 proteins (B-E; Y154H, R160W, R184C, G204D, and C206F respectively). Briefly, we evaluated the formation of H-bonds between residues of interest and surrounding residues throughout the simulation trajectory. For each variant, the molecular simulation was performed three times. Respective probability distributions of H-bonds are presented below each plot, offering insight into the differences in H-bond-forming capabilities between WT and protein variants.

REFERENCES

Ren J, Wen L, Gao X, Jin C, Xue Y, Yao X. 2009. DOG 1.0: illustrator of protein domain structures. *Cell Res* **19**: 271-273.

Zheng W, Sha QQ, Hu H, Meng F, Zhou Q, Chen X, Zhang S, Gu Y, Yan X, Zhao L et al. 2022. Biallelic variants in ZFP36L2 cause female infertility characterised by recurrent preimplantation embryo arrest. *J Med Genet* **59**: 850-857.
